# “I Climbed a Fig Tree, on an Apple Bashing Spree, Only Pears Fell Free”: Economic, Symbolic and Intrinsic Values of Plants Occurring in Slovenian Folk Songs Collected by K. Štrekelj (1895–1912)

**DOI:** 10.3390/plants11030458

**Published:** 2022-02-07

**Authors:** Živa Fišer

**Affiliations:** Department of Biodiversity, Faculty of Mathematics, Natural Sciences and Information Technologies, University of Primorska, Glagoljaška 8, SI-6000 Koper, Slovenia; ziva.fiser@upr.si; Tel.: +386-(5)-6117662

**Keywords:** ethnobotany, folk poetry, plant symbolism, ritual plants, useful plants

## Abstract

In this study we examine the occurrence of plants and their symbolic, economic, and intrinsic values in Slovenian folk songs. We have analyzed songs published by the ethnologist Karel Štrekelj between 1895 and 1912. Of the 8686 songs studied, plants occur in 1246 (14%) of them. A total of 93 plant taxa were found, belonging to 48 plant families. Grapevine is the most frequently mentioned species, followed by rosemary, wheat, carnation, and lily. About half of the taxa belong to cultivated plants (52%), followed by wild plants (42%). Exotic plants (i.e., not growing in the area) are mentioned only occasionally (6%). Half of all citations (49.3%) refer to the symbolic values, such as religion, love, death, economic status, or human qualities. More than a third of the citations (36.7%) are associated with plant’s usefulness, especially consumption, while only a small percentage of citations (14.0%), relate to environmental representation. Several verses show how our appreciation of some plants, especially those used as food, has changed over the centuries. Folk songs have turned out to be interesting sources of information, and although they cannot be fully trusted as historical documents, they can still be used as sources for understanding the relationship between people and plants.

## 1. Introduction

Traditional knowledge about plants, including their economic and symbolic values, is passed down from generation to generation in many different ways, such as by being written and via the oral tradition. Literary texts and poems across the globe, from the earliest times to the present, abound with plant references [1,2,3] and emphasize the past and present importance of plants in daily life. Although these texts cannot be fully trusted as historical documents [4], they can still be used as sources for understanding the relationship between humans and plants. Moreover, comparing plant occurrences in historical and contemporary literary texts can also reveal changes in plant symbolism and use over time and in different places.

Similarly to other literary documents, traditional folk songs also offer an interesting insight into the relationship between humans and plants. Traditional folk songs are typically studied within the domain of ethnomusicology, which focuses on how songs are performed and transmitted and the role of music in building cultural identities [5], with an emphasis on indigenous peoples and local communities. However, the study of songs from an ethnobiological perspective offers a different perspective on the same topic, focusing on the relationship between humans and nature. As several works have shown, traditional songs can serve as repositories of indigenous peoples’ ethnobiological knowledge [6,7,8], management practices [9], linguistic expressions (e.g., archaic words; [10]), and many other cultural values.

We can speculate that folk songs contain the most important, or in some cases the most widespread, plant species in local communities that have acquired symbolic, economic, and ecological values over time. The fact that folk songs and tales were passed down orally from generation to generation allowed them to change and adapt to local characteristics over time and in different regions. In regions where different ethnic groups, cultures, or religious beliefs have met, this has led to the blending of beliefs and practices.

Since their settlement at the present site in the 8th century AD, the Slovenes (then called Carantanians) lived in an area with a vibrant political and cultural history, in a region often referred to as “where the Orient meets the West”, both culturally and geographically [11]. The invaders brought with them plant and animal species, traditions, knowledge, and religious beliefs that merged with the existing cultures, forming a pagan-Christian mixture that can still be observed today in some festivals, such as the Slavic mythological figure of “Zeleni Jurij” or Green George, who is celebrated on St. George’s Day in April. 

In this work, we have analyzed Slovenian folk songs in order to understand how important plants were in the daily life of the predominantly peasant population in past centuries. In addition to representing a heritage on its own, folk songs may contain many more pieces of hidden information. They may reveal some long-forgotten practices in plant uses; they may also, for example, reveal what was the local people’s attitude towards native plants that gradually became the basis of plant conservation.

We hypothesize that the common native plant species and the species cultivated for consumption will represent the majority of the plant references. We expect the national flower and tree—carnation and lime—to be among the most frequently mentioned taxa in folk songs.

## 2. Results and Discussion

Plants are common in Slovenian folk songs, which underlines their importance for the local population. Out of 8686 songs, we found 1854 plant references in 1246 songs, which corresponds to 14% of all songs. A total of 97 plant taxa were recorded: 93 taxa belonging to 48 families of seed plants and three taxa of ferns and mosses. 77 taxa were identified to species level. The list of taxa and families (or higher taxonomic categories) can be found in Table 1 and in Supplementary Materials (Supplementary Material S1 and Table S1), where all categories assigned to species or higher taxa are also listed.

A similar study was conducted by Herrero and Cardaño [8] who studied folk songs from Castile and León (Spain). The proportion of songs with plant references in Slovenian folk songs (14%) is statistically lower than that found by [8] in songs from Castile and León (18%; *p* < 0.0001), as is the number of plant taxa found in songs (96 vs. 150 for Slovenia and Castile and León, respectively; *p* < 0.0001). However, such comparisons are difficult to comment on, as they depend strongly on the amount of material studied, the size of the area studied, and its species diversity. The number of Slovenian songs analyzed was higher than that of Castile and León (8686 and 7120, respectively), but on the other hand, the surface and plant diversity of Castile and León are higher. The area of Castile and León is almost five times larger than the area of present-day Slovenia, and with about 4000 flowering plants, Castile and León has a greater diversity of plant species than Slovenia. Moreover, the Spanish Empire’s trade with America [12] and other continents led to the importation of many useful and ornamental plants, which are also mentioned in the analyzed Spanish folk songs (peanuts, cocoa, cinnamon, cloves, tea, etc. [8,13]), while they are completely absent from Slovenian folk songs. However, the percentage of cultivated, wild and exotic (not growing in the area studied) taxa is surprisingly similar.

The average number of plant taxa cited per song is 1.5, which is significantly lower than in songs of Castile and León (1.7; *p* < 0.0001). The highest number of plant citations in Slovenian songs was 10, while for Castile and León it was 17 [13]. However, the most frequently cited plant taxa are surprisingly similar: rose, grapevine, carnation, wheat (*Triticum aestivum* L.), olive (*Olea europaea* L.), and laurel (*Laurus nobilis* L.) for Castile and León and grapevine, rosemary (*Rosmarinus officinalis*), wheat, carnation, lily (*Lilium candidum* L.), and lime for Slovenia. Most of these taxa are cultivated and are important features in many European cultures, especially in southern Europe. The importance of the rose is probably related to the same religion that Slovenia and Spain share, as the species is a very important symbol in Christianity [14,15]. Grapevine and wheat are also among the most important crops in Europe, so it is not surprising that they play such an important role in songs. However, a closer comparison of songs from Slovenia and Castile and León shows that Spanish songs contain many more species adapted to milder conditions, such as the Mediterranean olive or laurel, or the orange (*Citrus sinensis* (L.) Osbeck) and lemon (*Citrus limon* (L.) Osbeck), while Slovenian songs contain more temperate plants (e.g., *Fagus sylvatica, Picea abies, Abies alba).* The higher number of exotic taxa in Spanish songs can be explained by the more intense trade with other continents in the past. Of the exotic plants in songs from Castile and León, only coffee (*Coffea arabica* L.), black pepper (*Piper nigrum* L.), frankincense (*Boswellia sacra* Flueck.), and myrrh (*Commiphora myrrha* (Nees) Engl.) also appear in Slovenian folk songs, while tamarind (*Tamarindus indica*), cinnamon (*Cinnamomum cassia* Siebold), cloves (*Syzygium aromaticum* (L.) Merr. and L.M. Perry), palms (fam. Arecaceae), bamboo (*Phyllostachys* spp.), cocoa (*Theobroma cacao* L.), and tea (*Camellia sinensis* (L.) Kuntze) are never mentioned.

Rosaceae is the plant family with the highest number of taxa occurring in Slovenian folk songs (10 taxa), followed by Poaceae, Leguminosae, and Compositae, all represented by 6 taxa (Table 1). Rosaceae, Poaceae, and Leguminosae are among the most economically important plant families [16], with many edible representatives. Most taxa from these families mentioned in folk songs are used for consumption, but have also acquired symbolic value because of their importance (e.g., apple, rose, wheat). Four taxa were found in Lamiaceae, Betulaceae, and Brassicaceae. Other plant families are represented by three or fewer taxa. Ferns and mosses are also mentioned, but only with the common name “praprot” (Slovenian for fern) and “mah” (moss). The only exception, where a higher taxonomic category is mentioned, is peat (*Sphagnum* sp.), which is mentioned under the Slovenian name “šota”.

**Figure 1:**
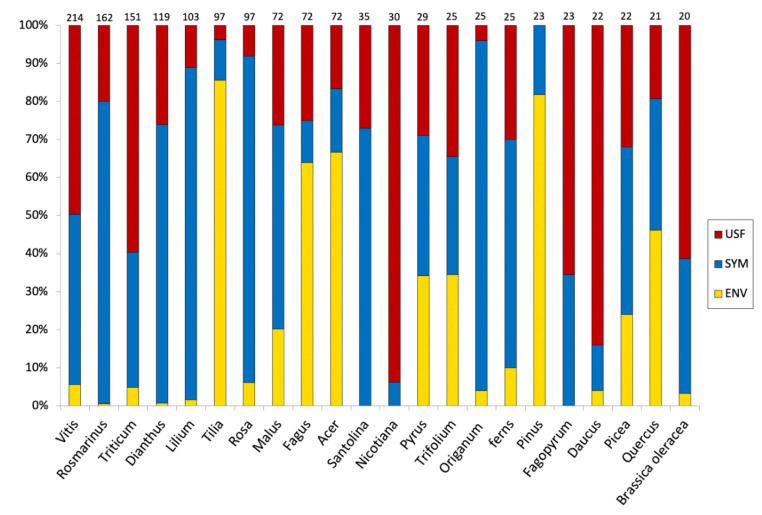
Plants occurring in Slovenian folk songs with 20 or more citations. The color of the bars represents the percentage of citations according to their value: USF—useful plant; SYM—plants with a symbolic value; ENV—plants as features of the environment. The number of songs containing the referenced taxon is written above each bar.

Grapevine is by far the most common plant species in Slovenian folk songs, appearing in 214 songs and accounting for 11.5% of all plant citations (Figure 1). This is not surprising, as viticulture and wine production have existed in this region for a long time and are of great economic and cultural importance to the Slovenian population. This led to an abundance of folk songs dedicated to wine. In the analyzed four-volume edition, an entire book section entitled Drinking Songs is dedicated to wine, containing 651 songs (Š5468–Š6119). Even the Slovenian national anthem by the poet France Prešeren, entitled “A Drinking Song” (in Slovenian “Zdravljica”), is dedicated to wine. Grapevine is followed by rosemary (162 songs, 8.7%), wheat (151 songs; 8.1%), carnation (119 songs; 6.4%), lily (103 songs; 5.6%), lime (97 songs; 5.2%), and rose (97 songs; 5.2%). These taxa account for over 50% of all plant references. Interestingly, rosemary, lilies and carnations are native to more southern parts of the Mediterranean and occur on Slovenian territory as a cultivated species. However, they have gained popularity throughout Europe and have become an important part of several European cultures. The carnation has even become the national flower of Slovenia.

Surprisingly, some very common annuals and herbaceous perennials are rarely or never mentioned in folk songs, despite their decorative and useful qualities. One of the most common and conspicuous field flowers is the ox-eye daisy (*Leucanthemum vulgare* (Vaill.) Lam.), while its smaller relative, the common daisy (*Bellis perennis* L.), is often found in lawns. Neither of these species are mentioned in the folk songs analyzed. The songs often mention white, yellow, red, and blue flowers without giving a specific name. For example, red flowers could be attributed to various Fabaceae, Compositae, Dipsacaceae, and Orchidaceae species; yellow ones to the species of Ranunculus, dandelions, or other representatives of the Compositae; blue ones to violets (*Viola* spp.), meadow sage (*Salvia pratensis*), forget-me-not (*Myosotis* spp.), or various Campanulaceae; and white ones to yarrow (*Achillea millefolium*), lily-of-the valley (*Convallaria majalis*), or various species of the Apiaceae family. Early spring flowering plants that indicate the end of winter, such as snowdrops (*Galanthus nivalis* L.), primrose (*Primula vulgaris*), spring crocus (*Crocus vernus* (L.) Hill), or black hellebore (*Helleborus niger* L.), are also surprisingly not represented in the songs. Cardaño and Herrero [13] also found that some common and conspicuous species (e.g., *Cistus* spp.) are rarely mentioned in songs, while those of greater importance to the habits and customs of the region (e.g., orange and lemon) are mentioned in greater numbers.

Finally, Slovenian folk songs rarely mention medicinal, poisonous, or edible wild plants. This was unexpected, as traditional medicine and wild plant gathering were widespread in the area [15]. Nowadays, several wild plants are widely collected as spices. Among the most commonly collected are the leaves of dandelions and wild garlic (*Allium ursinum* L.), the shoots of wild asparagus (*Asparagus acutifolius* L.) and black bryony (*Tamus communis* L.), the tops of Norway spruce, and the inflorescences of elder (*Sambucus nigra* L.), to name a few [17]. None of these species are mentioned in songs, at least not as food or condiments.

**Figure 2:**
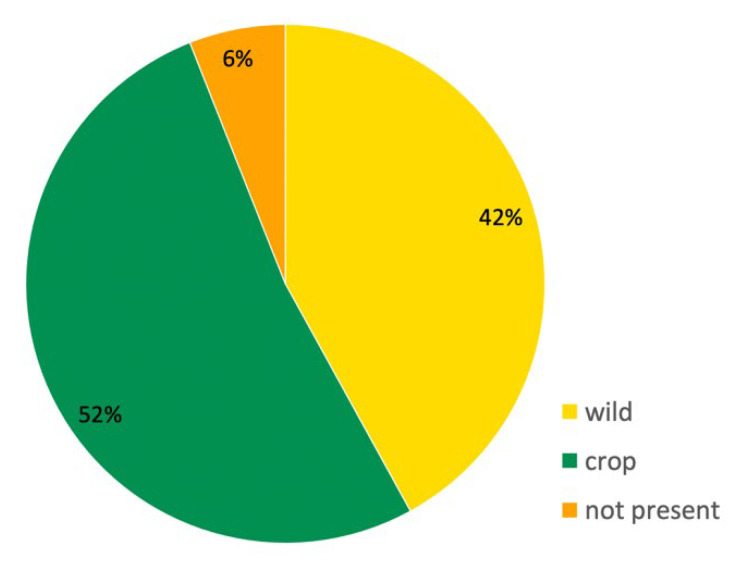
Percentage of wild, cultivated (crop), or exotic plant taxa cited in the analyzed folk songs.

Over half of the mentioned taxa (52%) belong to cultivated plants (Figure 2); most of them are cultivated for consumption (grains, beans, vegetables and fruit trees, including grapevine) or for ornamental purposes (e.g., carnation, rose, lily). The remaining taxa are represented by plants that grow in the wild (42%) and to exotic species that do not grow in the area (6%). Most of the cited wild species are trees or shrubs; among them, lime, beech, and maple are the most common. Only a few native species are annuals or herbaceous perennials, and some of them are cited only once or a few times: dandelion, spring gentian (*Gentiana verna* L.), lesser periwinkle (*Vinca minor* L.), or sorrel (*Rumex obtusifolius* L.). Clover (probably *Trifolium repens* L. and/or *T. pratense* L.) is among the most often mentioned native herbaceous plants.

Lastly, 6% of references belong to exotic species that do not grow in the region but are imported, or they are mentioned symbolically. Among the exotic species are the carob (*Ceratonia siliqua* L.)*,* orange, coffee, black pepper, and ginger (*Zingiber officinale* Roscoe). The carob tree is a legume originating in the Mediterranean region and is nowadays planted elsewhere (also in Slovenia) for ornamental purposes. It is possible that some trees were also cultivated in the past, most probably in the coastal region with a milder climate. Myrrh and frankincense are mentioned in several religious songs and refer to the gifts offered to the infant Jesus by the Three Wise Men. References to exotic fruits (oranges) might also originate from the fact that in the past many Slovenian women went to work abroad, mostly as wet nurses and governesses to Egypt (the so-called Aleksandrinke): “*The bird sings, the bird sings/In a beautiful green orange tree*” (“*Vtica poje, vtica poje/V lepoj zelenoj naranči*” Š951).

## 3. Classification of Plants According to Their Values

Although none of the plants were assigned to one category exclusively (Supplementary Material S1, Table S1) many plants are classified predominantly to one: rose, marjoram (*Origanum majorana* L.), and lily are mostly used as symbols, while lime and pine trees are most often cited to depict the environment. Tobacco (*Nicotiana tabaccum* L.), carrot (*Daucus carota* L.), or oats (*Avena sativa* L.) are plants mentioned exclusively for consumption; the first for smoking and chewing, while the latter two as food. Only a few plants (pear (*Pyrus sylverstris* L.), clover, oak (*Quercus* spp.), Norway spruce, and apple (*Malus domestica* Borkh.) are cited almost equally in all three categories.

**Figure 3:**
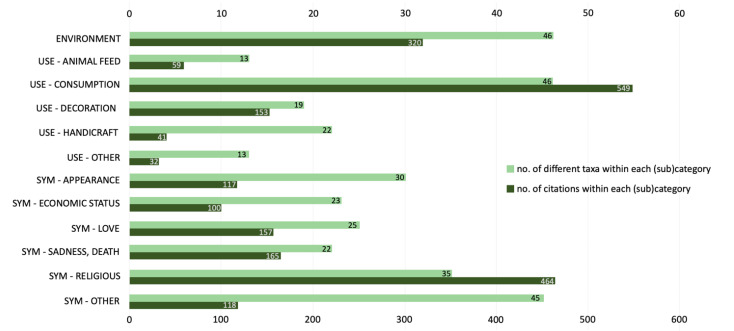
Number of different taxa (light green) and citations (dark green) within each (sub)category.

References with a symbolic meaning are by far the most common ones, representing almost half (49.3%) of all occurrences. Over a third of references (36.7%) are associated with their usefulness, while only a small percentage of plant references (14.0%) refer to the depiction of the environment. The number of different taxa and number of citations within each (sub)category in presented in Figure 3.

### 3.1. Plants with a Symbolic Value

#### 3.1.1. Religious Symbolic Values

In many folk songs with religious content, plants symbolize Mary, Jesus, St. Joseph, or angels: “There is a garden by the road,/In the garden grow some flowers./The first flower is a lily,/Because Mary is merciful/The second is a rose,/Because Mary is gentle./The third is rosemary,/Because Jesus is Mary’s son./The fourth is a carnation,/For the angels from heaven./The fifth is marjoram,/Because Jesus is Mary’s throne./I will gather them all,/And give them in honor of Mary.” (Pri cesti stoji garteljček,/V njem pa rastejo rožice./Ta prva rožca lilija,/Ker je Marija usmiljena./Ta druga roža gartroža,/Ker je MArija cartana./Ta tretka roža rožmarin,/Ker Jezus je Marijin sin. Ta šterta roža nageljnček./Kar so nebeški angeljčki./Ta šeta roža je mar’jon,/Ker Jezus je Marijin tron./Vse te rožce bom vkup pobral,/Mariji v čast jih bom dar’val.” Š4919). It is clear from this song that some plant references are used exclusively to form the rhymes (in Slovenian) and, thus, some of these do not have necessarily a true symbolic value. In the traditional songs analyzed, lilies and roses are the flowers most frequently associated with Mary, which is consistent with representations in visual art in Europe since the Middle Ages [14,18,19,20]. Some other flowers are also used in this context, but less frequently. Among lilies, the species that symbolically represents Mary is usually the white-flowered Lilium candidum, which was also given the name Madonna lily and has been associated with the Annunciation of the Blessed Virgin Mary since the Middle Ages [19,20].

Wheat and grapevine are also very often associated with religion, symbolizing the Eucharist: “*...The first flower/Is the yellow wheat:/At Holy Mass they make/Sacramental bread of it.../The second:/Is the beloved grape:/At Holy Mass they make/The Blood of Christ from it...*” (“*Perva rožica je leta:/Oj rumena všeničica:/Per svetej maši jo nucajo/Za samo sveto hoštijo.../Druha rožca je leta:/Ljuba vinenska tertica:/Per svetej maši jo nucajo/Za samo sveto rešnjo kri...*” Š4927).

##### Love

In the songs analyzed, love is usually represented by ornamental plants, especially rosemary, carnation, rasp-leaf pelargonium (*Pelargonium radens* H.E. Moore), marjoram, and cotton lavender, while native plants are rarely associated with love. Several of these flowers form the traditional Slovenian love bouquet (in Slovenian, “ljubezenski pušeljc”) [21,22,23]. This bouquet was an important part of daily life in the past. The carnation, always red in color, symbolizes life and love, not only in Slovenian folklore, but also in many other (especially Southern European) cultures [8,24]. Rosemary, which retains its fragrance both fresh and dried, represents faith, while the fragrant green rasp-leaf pelargonium represents hope. Therefore, the values represented in this bouquet are love, faith, and hope. Girls gave their boyfriends bouquets of carnations and rosemary (and sometimes other flowers) to symbolize their love and devotion.

##### Sadness and Death

Among the plants that symbolize negative feelings, death or sadness, or even criminal acts, many are not particularly attractive, such as clover: “*There grows green clover/in a green field./In the morning the farmer comes/and mows the clover.../Oh, sinner,/the same will happen to you.../In the evening you will lie down in your bed/all healthy and strong.../but then death will come to you/and knock you down*...” (“*Raste, raste detela,/Na zelenem travniku./Zautra kosec pride,/Jo doli pokosi.../Ravno, ravno tako/Boš grešnik ti.../Vzvečer doli ležeš,/Si frišek no si zdrav.../Kda ti smert do tebe pride,/Te doli pokosi...”* Š6120), or “*My mother asked me/“Where did you find this baby?”/“There, in those green ferns*” (“*Nas so mati prašali/“ Kje ste to dete najdeli?”/“Tam le v zeleni praproti*.” Š2278)), or have undesirable characteristics, such as the rush-producing nettle (*Urtica dioica* L.): “*She had three sons:/The first she threw into the sea/The second into the nettle*...”; (“*Sej je imela že sinke tri:/Enga je bla vrgla v morje,/Drugega je bla v koprivje..*.” Š171), the thorny blackthorn (*Prunus spinosa* L.), or the bitter wormwood (*Artemisia* spp.). Many of these plants, for example box (*Buxus sempervirens* L.) and Norway spruce, are evergreen. De Cleene and Lejeune [14] mention that in German speaking countries, several blue flowering spring flowers, such as violets, pyramidal bugle (*Ajuga pyramidalis*), bluebell (*Hyacinthoides non-scripta*), and the spring gentian (*Gentiana verna*), were believed to bring bad luck. In some European countries (e.g., Italy, Netherlands), periwinkle (*Vinca minor*) was used in children’s funeral wreaths or planted on graves (especially those of children), thus the Italian name Fiore di Morte (death’s flower) [15]. Indeed, the lesser periwinkle and spring gentian are associated with death also in some songs: “*Spring gentians faded,/they were dry and faded./Mila fell asleep among them;/never again did she wake up*.” (“Sp*anjšice ble ocvetele,/Ble so suhe že in vele./Mina je bla v njih zaspala,/Nikdar več ni z spanjšic vstala*.” Š234). Sadness or death is often represented by withering or dried flowers or plants (marjoram, spring gentian, rosemary).

Even the traditional bouquets given by girls to boys were not always associated with positive feelings. When boys went to war or when they died, girls made green bouquets that represented sadness or death: “*Last year you received/A bouquet of flowers/This year I will give you/A bush of nettle*...” (“*Vlan’ si še pušelc/Iz rožic dobil,/Pa letas germušelc/Ti dam iz kopriv*” Š4461). Rosemary and cotton lavender are usually mentioned as components of green bouquets. Carnations, roses, and lilies were also often associated with death, as they were among the few cultivated ornamental plants planted on graves.

The references to ferns are interesting and show the importance of ferns both as useful and symbolic plants. Ferns grow wild in nature and their symbolic meaning relates to nature. Since dry fern leaves were used by farmers in the past to stuff mattresses, they are usually mentioned in connection with poverty and/or sleep. Some poems mention that women gave birth in the ferns and left their unwanted children there. In other poems, women who could not have children found newborns in ferns and took them home. The roots of the worm fern (*Dryopteris filix-mas* (L.) Schott) were once used to cure intestinal worms [25], but also to induce abortions [26], which might explain the connection between ferns and infanticide. On a more positive note, ferns are also mentioned as the place where lovers secretly meet and make love.

##### Human Appearance and Characteristics

The specific properties of plants are often compared to humans, both with a positive and negative connotation. Female beauty was for example described through the comparison with red roses, white lilies, white buckwheat flowers, white and red poppies, and black berries of blackthorn: “*She was slim as hemp,/Red as a rose,/White as a poppy flower/God himself brought her to this world*!” (“*Je b’la tenka ko konoplja,/Rudeča kakor gartroža,/Bela kakor makov cvet,/Sam Bog te zvolil na ta svet!*” Š1036). Even male beauty was sometimes compared to flowers: “*My loved one is beautiful as carnation flower*” (“*Moj ljubi je lep/ko fajdelnov cvet*” Š2900) or “*My boyfriend is handsome as laurel flower*” (“*Moj šocel je lep/Kakor lomberjev cvet*” Š2907). However, in the latter case the beauty probably does not really refer to laurel flowers as they are rather inconspicuous, but flowers serve as symbol of the plant’s usefulness. Mosses, ferns, and other unattractive green plants or unappreciated vegetables are used to emphasize ugliness or stupidity: “stupid as a cabbage stem” (“*Per deli je terda/Kak zeljov kocen*” Š2926); “*Her face is green as moss/.../Everyone is scared of her*” (“*Zelena ku mah,/.../ Pa gleda ku sova,/Da je vsakig strah*” Š2887); or “If you want to be mine,/You have to buy some color,/So you won’t be as green,/As sorrel and absinthe...” (“*Če češ moj bit’,/Moraš barvo kupit’,/Da ne boš tko zelen,/Kot ta ščavlja in pelen*.” Š2654). However, the green and not particularly attractive hemp (*Cannabis sativa* L.) is usually associated with positive qualities, probably due to its many useful properties.

##### Economic Status

Several plants are used as symbols of economic status. Although some crops were regarded as “poor people’s food”, others symbolize richness or wealth. Potatoes, cabbage, beans, and turnips symbolize poverty: adjectives such as black (black potatoes-probably meaning burned potatoes), small (small beans) or stinky (stinky cabbage) are often associated with these foods: “*On Sunday there is nothing else (to eat)/Than a piece of meat/And some stinky cabbage*...” (“*V nedeljo ni druzga/Kot košček mesa/In zraven mav zelja/Usmrajenega*.” Š7399). Among the grains, wheat was the most appreciated one, while rye, barley and buckwheat are regarded as food of the poor or as animal feed: “*The pilgrims are gathering/To visit the holy mother by the lake./They are preparing food for the travel:/Wheat for the rich ones,/Barley for the poor ones*...” (“*Oj romarji se zbirajo/K mater božji na jezero./Oj za brašnjo napravljajo:/Ti bogati za pšenično,/Ti ubogi za ječmenovo*.” Š292). The low appreciation for barley is evident from several songs referring to eating barley porridge. Porridge was a common prison food and the saying “eating porridge” refers to being in prison [27].

#### 3.1.2. Useful Plants

##### Plants Used for Human Consumption

Grapevine is by far the most frequently referenced useful plant, used exclusively for consumption. Among the other fruits, the dominating species are temperate trees which have been cultivated in the region for centuries: apples, pears, hazelnuts, cherries, walnuts, plums, figs, sour cherries, olives, and peaches. Oranges are the only fruits that are not commonly grown in the region due to the mostly unsuitable climate. Nowadays, they are occasionally cultivated in the sub-Mediterranean part of Slovenia, but folk songs indicate that they were occasionally also grown in the past outside the warmer sub-Mediterranean: “*Grow, grow, orange tree,/Orange tree, noble tree*...” (“*Rasti, rasti pomoranča,/Pomoranča, žlahtno drev’!*” Š923).

Vegetables are mentioned less frequently than fruits. This is probably because growing vegetables was not very common before the 18th century; pumpkins, cucumbers, melons, turnip, carrots, radishes, onions, and garlic are among the few vegetables grown until the end of the 17th century [28]. Vegetables also often have a negative connotation, compared to fruits or grains, and represent a symbol of poverty or contempt for foreigners, e.g., “...*You will eat white turnip,/And sit hungry by the stove*…” (“*Belo repo bodeš jedla,/Lačna pol’ se k peči vsedla.*” Š8351) or “*Vlachs-farting peas, shitting lentils*!” (“*Vlah/Prdi grah,/Seri lečo*!” Š7718). Among the vegetables present in the region since the settlement of the Slavs (between the 6th and 8th century), only cabbage, turnip, and lettuce are mentioned in more than 10 songs, while lentils, faba beans, green peas, onions, radishes, and leeks are mentioned less frequently. Introduced vegetables and grains are mentioned only rarely (except potatoes and beans, which both appear in over ten songs), but are usually despised: e.g., “*On Friday we eat/Those damn beans:/When you put them in mouth/You almost vomit./On Saturday there is nothing else (to eat)/Than black potatoes,/How can I eat them/They smell like pitch*...” (“*V petek je tisti/Prokleti fižol:/V usta ga deneš,/Bi kmalu kozlal*./*V soboto ni druzga,/Kot črni krompir;/Kako ga bom jedel,/K smrdi kakor šmir!*” Š7402). None of the analyzed songs mention the now widespread bell peppers or tomatoes. Although they were introduced by the 19th century, they became more popular only in the beginning of the 20th century, after the analyzed songs had been recorded. In the last 100 years, those two vegetables became common and, together with some other new world vegetables, such as zucchini or eggplants, gained their place in traditional Slovenian cuisine.

Cultivated grains represent one of the most important food sources and the staple food of this region. Many cultivated grains are mentioned in folk songs, stressing their importance for the local population. Wheat is the most commonly cultivated and also the most appreciated grain species, which also resulted in the plentiful citations in folk songs. It was present in the region for at least 5000 years and Medieval times, taxes were paid in wheat [28]. The cheaper version of wheat is barley, one of the oldest and most cultivated crops worldwide. In Slovenia, barley is the main ingredient of the traditional porridge (in Slovenian language “ričet”). In the past, barley was even more common than today, as it was cheaper than wheat and, therefore, popular amongst the poorest: “*The pilgrims are gathering/To visit the holy mother by the lake./They are preparing food for the travel:/Wheat for the rich ones,/Barley for the poor ones*...” (“*Oj romarji se zbirajo/K mater božji na jezero./Oj za brašnjo napravljajo:/Ti bogati za pšenično,/Ti ubogi za ječmenovo*.” Š292). Folk songs also mention bread and pastries made from other grains, such as a special flatbread made of common buckwheat (“ajdova prosjača”) and bread made of oat or buckwheat. Buckwheat is second to wheat and is followed by oat. Other mentioned grains are proso millet, rye, and maize. Corn, now an important crop in Slovenia, is mentioned in only three songs. Corn was introduced in the 17th century and its cultivation progressed slowly in some regions as it competed with the much more popular buckwheat [11,28] and other cereals. By the end of the 19th century, corn was widely grown and its absence from songs is surprising.

Tobacco was used both for smoking and chewing, although all references except one refer exclusively to smoking: “I went to Celovec/.../I did not eat or drink anything/only smoked and chewed tobacco” (“V Celovc sem bil/.../Nič nisem jedel, nič ne pil,/.../Le rauhtabak in čiktabak/Je bila moja špiža.” Š7345).

##### Plants Used as Animal Feed

Fruits and other parts of plants are also mentioned as sources of food for wild and domesticated animals. The latter—horses, pigs, chickens—were fed with wheat, oats, clover, and hazelnuts. As a rather expensive commodity, wheat was used to feed noble horses, but the more common feed for working horses was clover or oats. Pigs were fed with acorns, beechnuts, and apples. Proso millet, but also grains of wheat, were used to attract wild birds. Mice are mentioned in a few songs, where they feed on proso millet, while in one song a bear is eating oats and lentils.

##### Decorative Plants

Among the decorative plants, most citations refer to the previously mentioned bouquets (see the section Plants with Symbolic Values: Love and Sadness and Death). Bouquets have been used throughout Europe for several purposes and on different occasions; the composition of flowers usually reflected their purpose e.g., [29,30,31]. Although none of the species that composed the traditional Slovenian bouquets are native in the region, they became an essential part of the Slovenian culture. Rosemary is the second most referenced plant species in Slovenian folk songs, which gained its popularity due to its scent and evergreen character; while carnations, as mentioned previously, even became the national flower symbol, as in some other European cultures (Spain, Monaco) [14:475]. These species were grown in gardens and pots around houses, on windowsills and balconies.

Rosemary has been an important plant species in the Mediterranean since at least Ancient Greece and was used later by both pagans and Christians [32]. Rosemary has many symbolic meanings in Europe, such as love, death, eternity, fidelity, virginity, and others (see [33] and references therein). Many of these symbolic meanings are also found in the Slovenian folk songs. Although rosemary is extensively used as a condiment nowadays, the references in folk songs are mostly symbolic ones and do not relate to its useful values.

Some songs mention the “German rosemary” instead of “rosemary”. The Slovenian name of *Santolina chamaecyparissus* L. is “nemški rožmarin” (translated into English as German rosemary), and in a few cases it is difficult to understand whether the reference relates to *Rosmarinus* or *Santolina*. Among the wildflowers, blue or white violets were also collected for bouquets.

Lilies and roses were (at least initially) not cultivated in gardens, therefore, they were not used in bouquets or wreaths but were often planted on graves. A special kind of religious bouquet, called “Mary’s bouquet”, was devoted to Virgin Mary and was composed of her symbol, the rose. Lilies used in bouquets were almost exclusively white, while roses were always red.

##### Plants Used for Handicrafts

Branches and trunks of various shrubs and trees were used to make objects of daily use. The songs mention the use of wood of Norway spruce for boats, silver fir for boards, apple for chests, lime and maple for cradles, hazel and dogwood for sticks, maple for musical instruments and arches, box for smoking pipes, juniper and alder for agricultural tools, such as hoes and plows, and oak for smaller decorative items such as Jesus crosses. Pliable branches of hazel and willow and stems of old man’s beard (*Clematis vitalba* L.) are mentioned as sources for making reins, baskets, or for tying: “*He came to get gbanca (traditional pastry),/It’s made of barely/And tied with a hazel branch*” (“*Po gbanco je prišel,/Ječmenova je,/Z ‘no leskovo trtico/Zvezana je.”* Š3581). The making of homemade baskets from hazel and willow branches is well documented from some Balkan countries [34,35] and was also an important source of income in some Slovenian villages [36]. Nowadays, basketry is practiced only on a small scale, but its ethnological importance was confirmed by the inclusion of weaving in the Register of Intangible Cultural Heritage [37]. Although hazel and willow baskets are still made for commercial purposes, baskets made from old man’s beard were only used for domestic purposes [36]. Textile fibers were obtained from herbaceous plants, such as hemp, flax, and stinging nettle. Similarly to the now commercially more important flax and hemp fibers, nettle fibers were used to make textiles in Central Europe before the introduction of cotton in the 19^th^ century, but their production ceased during World War II when other, cheaper fibers became more readily available [38].

##### Other Miscellaneous Uses

The folk songs studied also contain sporadic references to some medicinal and magical plants or plants used in religious rituals. Dafni et al. [33] classify ritual plants as plants or their parts used in private or official ceremonies to create a tunnel with gods or supernatural forces. These plants may include sacred trees, hallucinogenic and narcotic plants, incense, and aromatic plants with other uses. Religious uses mentioned in folk songs include sprinkling the dead with evergreen plant twigs and making funeral wreaths. These consisted of green plants, usually rosemary and also cotton lavender (*Santolina chamaecyparissus*), but were sometimes combined with colorful flowers, as mentioned in one of the folk songs: “*When I die/I will have a beautiful wreath/Of rosemary/And red carnations*” (“*Če jez dekle umrla bom,/Venček lep imela bom:/Z’ rožmarina blagiga,/Z’ nagelna rudečiga.*” Š6250).

The sprinkling of the dead with (usually evergreen) plant shoots at funerals has its origins in antiquity and has survived as part of the Christian burial tradition. Various plants, usually evergreens, were used in burial rituals, such as myrtle, basil, olive tree, spruce, juniper, rosemary, box, and others [33,39,40,41]. In reports from some countries (e.g., Iraq), the evergreen habit is said to preserve the soul and is referred to as evergreen life force [42]. One of the folk songs says: “*She entered the room/And sprinkled him/With green rosemary*” (“*Prek praga je stopila,/Z zelenim rožmarinom/Ga poškropila*” Š205).

Only three plant species were mentioned as having medicinal properties, namely carrot, fennel (*Foeniculum vulgare* Mill.), and laurel, as well as the magical “koren lečen”. Koren lečen is a root of a presumably magical plant that cures all diseases: “*I have an unknown taproot,/Unknown taproot, medicinal taproot (koren lečen):/I put it under my tongue,/In the evening I get very sick/In the morning I lie there dead/.../The next day the people in the castle cry/.../Only the court jester laughs,/Talks and says:/“I have a strong feeling/That the young Zora is not dead./.../He (the king) opens my mouth/and removes the root/Unknown root, healing root (koren lečen)/*...” (“*Sej imam jest neznan koren,/Neznan koren, koren lečen:/Ki ga pod jezik položim,/Precej zvečer hudo zbolim./Zjutraj pa že mertva ležim./.../Le grajski norec se smeji,/Tako-le pravi, govori:/„Močno, močno se meni zdi,/De mlada Zora mertva ni./.../Pa Zori vzame ’z ust koren,/Neznan koren, koren lečen.*” Š114) or “...*He plucks a tap root/a dear and noble one:/.../If you, my darling, are better,/this (taproot) will cure you;/But if you are dying,/you will be much worse with it./*...” (“*Vun potegne žlahten koren;/.../Če tebi k zdravji bo,/Potem ti preci bole bo;/Če pa tebi smrti bo,/Potem ti taki huje bo!*” Š129). Although there has been debate in the past as to which plant species “koren lečen” refers to, and some candidates have been mentioned, such as bryony (*Bryonia* L.) [43] or blessed thistle (*Cnicus benedictus* L.) [28], the most common opinion is that it is a fictional magical species.

Fennel is mentioned as a remedy to ward off snakebites: “...*Eat, eat the fennel,/so that the colorful snake will not bite you*...” (“*Jej, jej, jej, koromač,/Da te ne piči pisan kač!*” Š7851). This song was sung by children at Easter while eating fennel and other blessed Easter foods. In one of Pliny’s works, he mentions a fable about snakes eating fennel when they shed their skin to improve their eyesight. This fable led to the development of an ointment made from viper skin, fennel, and frankincense to improve eyesight [44]. The use of fennel seeds as an antidote is reported from Hindu and Chinese cultures [45].

The absence of medicinal and magical references in Slovenian folk songs was unexpected, as traditional medicine and wild plant gathering are widespread in the area [15] and Mlakar [28] lists a large number of plants with apotropaic magical powers used in Slovenia. However, a similar observation was made by Cardaño and Herrero [13] and De Cleene and Lejeune [14]. The latter found only five references to medicinal use in folk songs of Castile and León (Spain), and, surprisingly, none of the cited species (lemon, orange, apple and rose) is primarily associated with medicine.

#### 3.1.3. Plants as Features of the Environment

The most frequent plants used to describe the environment are native or cultivated trees: lime is by far the most cited tree, followed by maple, beech, pine, grapevine, apple, pear, and oak. This comes to no surprise, as lime is regarded as the most important tree species in the Slovenian culture. The importance of lime has roots in the Slavic times, when lime was worshiped as a ritual tree. Thus, since the early days, lime trees were planted in village centers, where all important events took place: “*There is a village named Dolina/In the middle there stands a lime tree/Underneath the gypsies gather*...” (“*Stoji, stoji Dolina vas,/Na sred vasi pa lipica./Se tam cigani zbirajo*...” Š133). This tradition is still vivid today in Slovenia and large lime trees are often protected by law as sites of cultural and natural heritage [46]. Of course, lime is not the only tree species that was planted in villages, but was sometimes replaced by oaks, pines, pears, and other trees: “*There is a green pine growing in the courtyard/a black horse is tied to it...*” (“*Na dvori vam zelen bor,/Zanj’ privezan konjič vran..*.” Š4746) or “*A pear is growing in front of the house/Underneath the pear is a cool shade..*.” (“*Pred vrati vam hruška zrasla,/Pod njo vam je hladna senca*...” Š5083). Some tree species, such as beech, willow, or pine, are usually used to describe the natural environment outside the human settlements: “*The shepherd is herding goats/In the green pinewood…*” (”*Kazarič mi kazice pase/U zeljanen borawji*...” Š176).

Among the herbaceous plants used to describe the environment are both wild plants (e.g., clover, ferns, stinging nettle, violets), as well as cultivated ones (e.g., wheat, roses, cotton lavender, lilies).

## 4. Materials and Methods

### 4.1. Area of Study

Slovenia is located in Central Europe, bordering Italy to the west, Austria to the north, Hungary to the northeast, and Croatia to the south and east, including also a 43 km long stretch of the Adriatic Sea in the southwest. The country covers an area of 20,273 square kilometers and has a population of 2.06 million, with expatriate communities of Slovenian or mixed origin also living in all neighboring countries. The population density is 101 inhabitants per square kilometer, which is similar to neighboring countries, but low compared to most Western European countries.

The climate in the central and eastern parts of the country is temperate. The western and southwestern coastal parts are influenced by the Adriatic Sea and have a mild sub-Mediterranean climate, while the Alps in the north dictate an alpine climate. A large part of the country (almost 60%) is covered by dense forests, in which the beech (*Fagus sylvatica* L.) is the most common tree species. In the Alps, evergreen coniferous forests of spruce (*Picea abies* (L.) H. Karst.) and fir (*Abies alba* Mill.) are found, while the vegetation of the sub-Mediterranean climate is dominated by deciduous forests of downy oak (*Quercus pubescens* Willd.) and common (*Carpinus betulus* L.) and oriental hornbeam (*C. orientalis* Mill.). Slovenia has a rich plant diversity due to its location at the intersection of climatic and geological factors. Altogether, 3119 plant taxa (seed plants and ferns) are present [47].

In 2010, 474,432 hectares were used for agriculture. This corresponds to 23.4% of the area of Slovenia. A large part of the agricultural land is permanent grassland and pastures (58%), followed by arable land (36%) and permanent crops (6%). The trend in agricultural land use is declining, with a decrease of 2% between 2000 and 2010 [48]. In past centuries, agriculture was much more important and more land was used for it, mostly divided among small farms.

### 4.2. Data Collection

**Figure 4:**
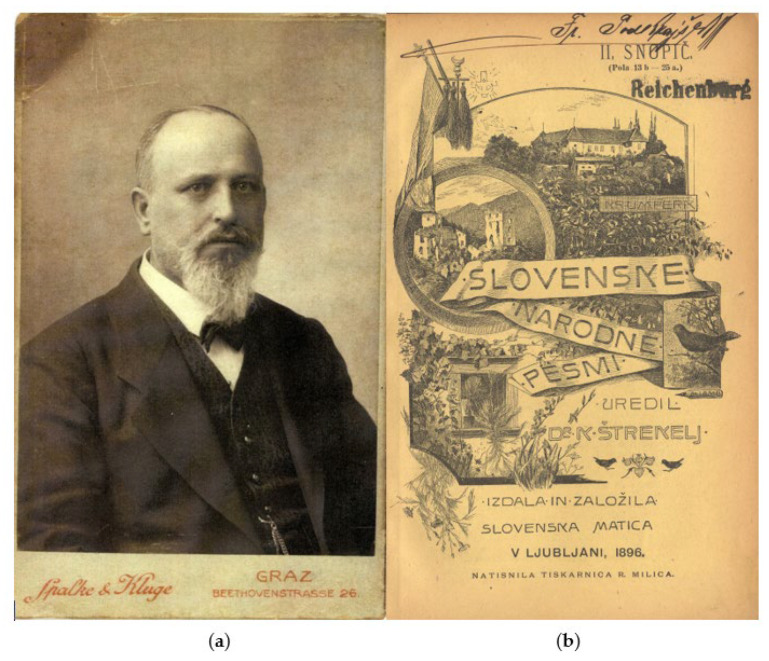
(**a**) Photograph of Karel Štrekelj from the period when he was teaching Slavic philology at the University of Graz; (**b**) and the title page of the second volume of the Slovenian folk songs (right).

In this study, we have analyzed 8686 Slovenian folk songs collected by the ethnologist Karel Štrekelj (Figure 4a) and published in 14 notebooks between 1895 and 1912 (Figure 4b) [49,50,51,52]. His work was completed posthumously by his student Jože Glonar, who edited two more notebooks (Notebooks 15 and 16), published in 1923 [53]. The collection is considered the most comprehensive Slavic collection of folk literature of its time, and Štrekelj’s work was distinguished by its scientific methodology, which was recognized by several foreign critics of the time [54]. The songs were transcribed in their dialects rather than converted into literary language, and all songs indicate geographical origin. Although Štrekelj encountered the problem of songs with a known author and songs “in transition” to folk songs and first resisted publishing these songs because, in his opinion, they contradicted the rules of folklore and folk poetics, in the end he decided to compromise and published a supplement of these “non-folk” songs in the 3rd and 4th editions [54]. Štrekelj divided the songs into several categories and subcategories (references to songs throughout the article are marked with a unique code composed of the letter Š and a number corresponding to the number of the song in Štrekelj’s four-volume edition, namely Š1–Š8686): narrative songs (Š1–Š1006), love songs (Š1007–4729), songs for special occasions (furtherly subdivided into ritual songs (Š4730–5180), dancing songs (Š5181–5249), marriage songs (Š5250–5467), drinking songs (Š5468–6199), funeral songs (Š6120–6400)), religious songs (Š6401–6732), songs about professions (Š6733–8538), and humorous songs (Š8539–8686).

During the analysis, each song was read and plants and products derived from plants (e.g., a juniper hoe; an alder plow; a buckwheat cake) were identified and compiled in a table. Along with the plant names, the passages were transcribed and various classifiers (see the “Data Classification” section) were assigned to each plant occurrence. When verses alluded to more than one use of a plant, we included the reference in multiple categories. In 2019, the whole collection has been published online and is now freely available in the Slovenian digital library dLib (Digitalna knjižnica Slovenije. Available online: www.dlib.si (accessed on 10 January 2022)) [55].

### 4.3. Plant Identification

The Slovenian language has numerous dialects, which can lead to polysemy (e.g., fajgln can be used for carnation (*Dianthus caryophyllus* L.) and wallflower (*Erysimum cheiri* (L.) Crantz)). In such cases, we selected the most plausible botanical species depending on the context. In many cases, different spellings and names were found for the same plant species (e.g., the Slovenian names ‘vrtnica’, ‘šipek’, or ‘gartroža’ are used for rose (*Rosa* spp.) in different regions; similarly, ‘trta’, ‘trs’, and ‘grozdje’ are used for grapevine (*Vitis vinifera*) or ‘ajda’, ‘ejdica’ or ‘hojda’ for buckwheat (*Fagopyrum esculentum* Moench)). Some plant names have changed over time and are no longer used. Species identification has, therefore, been difficult in some cases. Several written [47,56] and web-based sources [57,58] were compared to understand the plant name. In cases where identification was not possible, the plant reference was omitted (e.g., no explanation was found for ‘zizer’ and ‘mramurka’ and these references were included in the analysis as unidentified seed plants). However, for most plants, identification was straightforward. For each plant species or taxonomic category, its occurrence was recorded as growing in the wild, cultivated, or not present in the area (exotic species). The full list of plant taxa is given in Table 1, and all vernacular names as recorded in the songs and their values are explained in the Supplementary Materials. The scientific names of plants and higher taxonomic categories have been updated to the currently accepted names listed in World Flora Online [59].

### 4.4. Data Classification

Classification of plant occurrences was adapted from [4], who classified all plant references into three groups: 1. *Plants as features of the environment* (ENV); 2. *Useful plants* (USF); and 3. *Plants used as symbols* (SYM). According to the classification, one plant species can fit into more categories, depending on the poem. Moreover, one plant occurrence can also be classified into more than one category. Some examples of classifications of plant references into categories are mentioned below.

#### 4.4.1. Plants as Features of the Environment (ENV)

Plants or plant formations mentioned in folk songs may be mentioned simply as elements of the landscape, without any other visible significance. Trees, especially lime (*Tilia* spp.), but also maple (*Acer* spp.) and oaks (*Quercus* spp.), represent important cultural features in Slovenian villages and are, therefore, often mentioned in songs. Trees were (and still are) planted in the centers of villages and the inhabitants met there daily; dances and important meetings of elders also took place there, e.g., “*There is a lime tree/A green lime tree/Under the lime tree a table/A stone table*...” (“*Stoji, stoji lipa,/Lipa zelena;/Pod njoj stoji miza,/Miza kamena*...” Š4929).

#### 4.4.2. Useful Plants (USF)

In the past, plants represented one of the most important natural resources for humans. Plants and their parts have been used for: making shelters, furniture, and tools; for consumption and as medicine; and also for decoration. Because of the many different uses of plants, this category has been divided into several subcategories: (a) plants for human consumption (e.g., food, drink, and other consumption, such as smoking); (b) plants for animal feeding; (c) ornamental plants (e.g., bouquets, home decorations, and garden plants); (d) plants for handicrafts (e.g., wooden objects and other artifacts); (e) other (e.g., plants for religious rituals, medicinal plants).

#### 4.4.3. Plants with a Symbolic Value (SYM)

Similarly to most cultures around the world, the people of Slovenia assigned symbolic meanings to various plants. Because of the many different symbolic values, this category has been divided into several subcategories: (a) plants as religious symbols (e.g., in religious folk songs, plants are often used metaphorically to represent God, Jesus, the Virgin Mary, St. Joseph, or angels); (b) plants as symbols of love; (c) plants symbolizing negative feelings, sadness, or death (e.g., flowers given as gift at the departure of a loved one to war or plants that grow out of a grave); (d) plants as symbols of economic status (e.g., some edible plants are associated with wealth, others with poverty); (e) plants representing the appearance or characteristics of a person (e.g., beauty: “*beautiful like a carnation blossom*” (“*lep ko fajdelnov cvet*” Š2900); health: “*green (sick) like absinthe and sorrel*” (“*zelen kot ta ščavlja in pelen*” Š2654); size: “*tears as (big as) grapes*” (“*debele solze kakor vinske jagode*” Š15), color: “*red like a rose*” (“*rdeča ko gatroža*” Š369), and other qualities; (f) other.

## 5. Conclusions

Plants play an important role in Slovenian folk songs. Half of the mentions of plants have a symbolic value, followed by the representation of the environment and, finally, by their useful aspects. The analysis revealed that indigenous plants growing in the wild were mentioned much less frequently than those that were important for survival (cereals, grapevine) or from a cultural point of view (lime, carnation, rosemary). Interestingly, the songs revealed some interesting findings not only about the plants that appear in the songs, but also about plants that do not appear in them. Wildflowers are rarely mentioned in Slovenian folk songs, suggesting that our belief in a romantic coexistence of the peasant population with nature and their appreciation for nature may not be entirely realistic. Medicinal, poisonous, or edible wild plants also rarely appear in the songs. Folk songs have proven to be interesting sources of information, and, although they cannot be fully trusted as historical documents, they can still serve as sources for understanding the relationship between people and plants.

Although this work is a comprehensive study of Slovenian folk songs, it contains only a preliminary catalog of plants and their values. The availability of digitized data combined with mathematical approaches has led to the emergence of new fields that offer a multimodal network representation of data, such as computational gastronomy [60] or, in our case, computational folkloristics [61]. Such methods allow us to explore the complexity of a folkloric corpus, such as the folk songs presented in this article, at multiple levels of resolution, allowing for a more holistic interpretation of culture.

## Figures and Tables

**Table 1:** List of plants mentioned in the analyzed Slovenian folk songs.

Family Name	Scientific Name	English Name	Slovenian Name	Citations	Presence
Adoxaceae	*Sambucus nigra* L.	elder	bezeg	3	wild
Amaryllidaceae	*Allium ampeloprasum* L.	leek	por	1	cultivated
	*Allium cepa* L.	onion	čebula	4	cultivated
Apiaceae	*Daucus carota* L.	carrot	korenje	22	cultivated
	*Foeniculum vulgare* Mill.	fennel	koromač	1	cultivated *
Apocynaceae	*Vinca minor* L.	lesser periwinkle	zimzelen	1	wild
Asparagaceae	*Convallaria majalis* L.	lily-of-the-valley	šmarnica	2	wild
Berberidaceae	*Berberis vulgaris* L.	barberry	češmin	1	wild
Betulaceae	*Alnus* spp.	alder	jelša	6	wild
	*Betula pendula* Roth.	birch	breza	11	wild
	*Carpinus* spp.	hornbeam	gaber	2	wild
	*Corylus avellana* L.	hazel	leska	22	wild
Brassicaceae	*Brassica oleracea* var. *capitata* L.	cabbage	zelje	20	cultivated
	*Brassica rapa* var. *rapa* L.	turnip	repa	114	cultivated
	*Erysimum cheiri* (L.) Crantz	wallflower	zlati šebenik	1	cultivated
	*Raphanus sativus* L.	radish	redkev	3	cultivated
Burseraceae	*Boswellia sacra* Flueck.	frankincense	bozvelija	8	absent
	*Commiphora myrrha* (Nees) Engl.	myrrh	mira	9	absent
Buxaceae	*Buxus sempervirens* L.	box	pušpan	6	cultivated
Cannabaceae	*Cannabis sativa* L.	hemp	konoplja	17	cultivated
Caryophyllaceae	*Dianthus caryophyllus* L.	nagelj	carnation	119	cultivated
Compositae	*Artemisia* spp.	wormwood	pelin	11	wild **
	*Helianthus tuberosus* L. ^2^	Jerusalem artichoke	topinambur	1	cultivated
	*Helichrysum* spp.	immortelle	smilj	2	cultivated
	*Lactuca sativa* L.	lettuce	vrtna solata	12	cultivated
	*Santolina chamaecyparissus* L.	cotton lavender	nemški rožmarin	30	cultivated
	*Taraxacum officinale* agg.	dandelion	regrat	1	wild
Cornaceae	*Cornus* spp.	dogwood	dren	4	wild
Cupressaceae	*Juniperus communis* L.	juniper	brin	7	wild
Ericaceae	*Erica carnea* L.^1^	heath	spomladanska resa	3	wild
Fagaceae	*Fagus sylvatica* L.	beech	bukev	35	wild
	*Quercus* spp.	oak	hrast	21	wild
Gentianaceae	*Gentiana verna* L.	spring gentian	spomladanski svišč	1	wild
Geraniaceae	*Pelargonium radens* H.E.Moore	rasp-leaf pelargonium	roženkravt	10	cultivated
Juglandaceae	*Juglans regia* L.	walnut	oreh	13	cultivated
Lamiaceae	*Ocimum basilicum* L.	basil	bazilika	6	cultivated
	*Origanum majorana* L.	marjoram	majaron	25	cultivated
	*Rosmarinus officinalis* L.	rosemary	rožmarin	162	cultivated
	*Salvia officinalis* L.	sage	žajbelj	1	cultivated
Lauraceae	*Laurus nobilis* L.	laurel	lovor	5	cultivated
Leguminosae	*Ceratonia siliqua* L.	carob	rožičevec	1	cultivated
	*Lens culinaris* Medikus	lentil	leča	8	cultivated
	*Phaseolus vulgaris* L.	common bean	fižol	12	cultivated
	*Pisum sativum* L.	pea	grah	4	cultivated
	*Trifolium* spp.	clover	detelja	25	wild
	*Vicia faba* L.	broad bean	bob	5	cultivated
Liliaceae	*Lilium candidum* L.	Madonna lily	lilija	103	cultivated
Linaceae	*Linum usitatissimum* L.	flax	lan	3	cultivated *
Malvaceae	*Tilia* spp.	lime	lipa	97	wild
Moraceae	*Ficus carica* L.	common fig	figa	4	cultivated *
	*Morus* spp.	mulberry	murva	2	cultivated
Oleaceae	*Fraxinus excelsior* L.	ash	jesen	2	wild
	*Olea europaea* L.	olive	oljka	2	cultivated
Papaveraceae	*Papaver rhoeas* L.,	common poppy	poljski mak	1	wild
	*P. somniferum* L.	opium poppy	vrtni mak	8	cultivated
	*Papaver* spp.	poppy	mak	6	cultivated, wild
Pinaceae	*Abies alba* Mill.	silver fir	jelka	4	wild
	*Picea abies* (L.) H. Karst.	Norway spruce	smreka	22	wild
	*Pinus* spp.	pine	bor	22	wild
Piperaceae	*Piper nigrum* L.	pepper	poper	1	absent
Poaceae	*Avena sativa* L.	oat	oves	18	cultivated
	*Hordeum vulgare* L.	barley	ječmen	10	cultivated
	*Panicum miliaceum* L.	proso millet	proso	11	cultivated
	*Secale cereale* L.	rye	rž	6	cultivated
	unclassified Poaceae			50	cultivated
	*Zea mays* L.	corn	koruza	3	cultivated
	*Triticum aestivum* L. em. Fiori and Paol.	wheat	pšenica	151	cultivated
Polygonaceae	*Fagopyrum esculentum* Moench	buckwheat	ajda	23	cultivated
	*Rumex obtusifolius* L.	bitter dock	topolistna kislica	1	wild
Ranunculaceae	*Clematis vitalba* L.	old man’s beard	navadni srobot	1	wild
Rosaceae	*Fragaria* spp.	strawberry	jagoda	5	wild
	*Malus domestica* Borkh.	apple	jablana	72	cultivated
	*Prunus avium* L.	sweet cherry	češnja	11	cultivated
	*Prunus cerasus* L.	sour cherry	višnja	3	cultivated
	*Prunus domestica* L.	common plum	sliva	7	cultivated
	*Prunus persica* (L.) Batsch	peach	breskev	1	cultivated
	*Prunus spinosa* L.	blackthorn	črni trn	7	wild
	*Pyrus communis* L.	pear	hruška	29	cultivated
	*Rosa* spp.	rose	vrtnica	97	cultivated
	*Rubus* spp.	brambleberry	robida	3	wild
Rubiaceae	*Coffea arabica* L.	coffee	kava	13	absent
Rutaceae	*Citrus sinensis* (L.) Osbeck	sweet orange	pomaranča	6	absent
Salicaceae	*Populus tremula* L.	aspen	trepetlika	1	wild
	*Populus* spp.	poplar	topol	1	
	*Salix* spp.	willow	vrba	9	wild
Sapindaceae	*Acer campestre* L.	field maple	maklen	1	wild
	*Acer* spp.	maple	javor	34	wild
Solanaceae	*Nicotiana tabacum* L.	tobacco	tobak	30	cultivated
	*Solanum tuberosum* L.	potato	krompir	14	cultivated
Ulmaceae	*Ulmus* spp.	elm	brest	1	wild
Urticaceae	*Urtica dioica* L.	stinging nettle	kopriva	15	wild
Violaceae	*Viola* spp.	violet	vijolica	15	wild
Vitaceae	*Vitis vinifera* L.	grapevine	vinska trta	214	cultivated
Zingiberaceae	*Zingiber officinale* Roscoe	ginger	ingver	1	absent
	Unidentified seed plant			3	
	Bryophyta	moss	mah	4	wild
	*Sphagnum* spp.	peat moss	šota	1	wild
	Pteridophyta	fern	praprot	30	wild

* also wild, ** also cultivated. ^1^ could refer also (but unlikely) to *Calluna vulgaris* (L.) Hull. ^2^ Could refer also to *Solanum tuberosum* L.

## Data Availability

The songs analyzed in this study are openly available in the Digital Library of Slovenia in a PDF or TXT file format at the following link: https://www.dlib.si/details/URN:NBN:SI:DOC-U1XDVBQI (accessed on 16 January 2022).

## Supplementary Materials

The following supporting information can be downloaded at: https://www.mdpi.com/article/10.3390/plants11030458/s1, Supplementary Material S1: Selected examples of plant references in Slovenian folk songs, divided according to their environmental, symbolic or useful value. All vernacular names or different spellings used in Slovenian folk songs are listed. If the most commonly used Slovenian name does not appear in the songs, it is marked with an asterisk (*). English names are in parentheses. References to songs are marked with a unique code consisting of the letter Š and a number corresponding to the song in the four-volume edition of Slovene folk songs edited by Karel Štrekelj (e.g., Š1-Š8686); Table S1: Table showing the number of plant citations for each plant taxon within each (sub)category.
