# Supplementary material for: “I Climbed a Fig Tree, on an Apple Bashing Spree, Only Pears Fell Free”: Economic, Symbolic and Intrinsic Values of Plants Occurring in Slovenian Folk Songs Collected by K. Štrekelj (1895–1912)
Source: Plants (Basel). 2022 Feb 7;11(3):458. doi: 10.3390/plants11030458 (PMC8838053; doi:10.3390/plants11030458)
Supplement: Supplementary file 1 [file plants-11-00458-s001.zip › plants-1580314/plants-1580314 - Appendix S1.pdf]

plants

MDPI

Supplementary Materials

Appendix S1

Selected examples of plant references in Slovenian folk songs, divided according to their environmental, symbolic or useful value. All vernacular names or different spellings used in Slovenian folk songs are listed. If the most commonly used Slovenian name does not appear in the songs, it is marked with an asterisk (*). English names are in parentheses. References to songs are marked with a unique code consisting of the letter Š and a number corresponding to the song in the four-volume edition of Slovene folk songs edited by Karel Štrekelj (e.g. Š1-Š8686).

FUNGI goba (mushroom)
USF: *Consumption* "The old woman is harvesting mushrooms, / the old man is weeding carrots..." (Š7764); baking mushrooms (Š8331)

BRYOPHYTA mah (moss)
ENV: *Landscape* "Now is (the monastery) all covered with moss..." (Š86)
SYM: *Ugliness* green as moss (Š2287)

***Sphagnum* L.** šota (peat moss)
USF: *Economic status* "...Everybody knows the people from Morost: they know how to cut the peat..." (Š8544)

PTERIDOPHYTA praprot, praprota, prapret, praprotnek, praprod (fern)
ENV: *Landscape* giving birth in the ferns: "The mother asked her daughter: "Where did you have a son?" "Up there by St. Jacob, in the green ferns..." (Š1761); *Other* ferns have no flower (Š5152); name of a village (Š8606)
SYM: *Making love* Making love in the ferns: "You are beautiful, I am handsome, let's go cut the ferns..." (Š1929); *Hard work* cutting ferns with a young boy is better than counting money with an old man (e. g. Š0911); *Economic status* ferns growing on the field or in the house represent poverty: "Ferns are growing on the field, / hunger is jumping around the house!" (Š3533); *Religious* "His (John the Baptist) food was fern roots..." (Š5022)
USF: *Consumption* eating fern roots (Š5022); *Bedding* making a bed out of dry ferns (Š2155): *Other* Digging / cutting ferns (Š7766)

ADOXACEAE
***Sambucus nigra* L.** bezeg (elder)
SYM: *Beauty* "Elder wood in beautiful, white and green, but even more beautiful is being single, joyful and happy..." (Š1402)
USF: *Handicraft* pipe (Š8558)

AMMARYLLIDACEAE
***Allium ampeloprasum* L.** por (leek)
USF: *Consumption*

***Allium cepa* L.** čebula, čbula, luk (onion)
SYM: *Happiness* "Last year I weeded / Onion and poppy flowerbeds, this year I won't even weed stinging nettle..." (Š2280)
USF: *Consumption*

APIACEAE
***Daucus carota* L.** korenje, korenček, korejne, merkava, merkavca (carrot)
ENV: *Landscape* "There are three cabbages on the field, three cabbages and some carrots..." (Š5207)
SYM: *Poverty*; *Religious* "...St John... spent thirty-three years in the desert... / his food was wild carrots, his drink was morning dew... " (Š5021)
USF: *Consumption; Medicine* noble carrot given to a sick girl (Š0129)

***Foeniculum vulgare* Mill.** koromač (fennel)
USF: *Medicine* "...Eat, eat the fennel, so the colorful snake won't bite you..." (Š7851)

APOCYANACEAE
***Vinca minor* L.** zimzelen*, barbarik zeleni (lesser periwinkle)
SYM: *Death, sadness* periwinkle grew on a grave (Š0740)

ASPARAGACEAE
***Convallaria majalis* L.** šmarnica, konvalium (lily-of-the-valley)
SYM: *Lullaby* "Sleep, field lily, sleep lily-of-the-valley, small and beloved, sleep, my only son... " (Š8182); *Other* (Š82)

BERBERIDACEAE
***Berberis vulgaris* L.** češmin (barberry)
SYM: *Human characteristic* "She is like barberry, / against the others!" (Š2931)

BETULACEAE
***Alnus* sp.** jelša*, jevša, još, jos (alder)
ENV: alder stump (Š7789)
SYM: *Other* alder leaves (Š7874), alder country (Š0947)
USF: *Handicraft* plow (Š8608)

***Betula pendula* Roth.** breza, brezica, brezjiče, brez, brezje (birch)
ENV: making love between the birch trees (Š2245)
SYM: *Other* birch country (Š0947); *"The hornbeam is getting married, / He is marrying the longhaired birch...*" (Š0997); *Beauty* comparing the beautiful birch wood to the joy of being unmarried (Š1400)
USF: *Handicraft* reins: "...They plow with alder plows; they brake with birch reins..." (Š8608)

***Carpinus* sp.** gaber (hornbeam)
SYM: *Religious* "Unfortunate hornbeam shade, / where Jesus could not be born..." (Š0426); *Other* "The hornbeam is getting married, / He is marrying the longhaired birch..." Š0997)

***Corylus avellana* L.** leska, leščovje, lešniki, lešnikiči, lešenči (hazel)
ENV: hazel hedge "...Nothing else grows there, / only hazel trees..." (Š6747); a forest of slender hazel (Š0560); *Other* hazel country (Š0947)
SYM: *Gift* "Be mine, / I'll give you hazelnuts, / If you don't like dried ones, / I'll collect fresh ones." (Š2620)
USF: *Consumption*; *Handicraft* stick (Š0307); twig used to tether (Š3581)

BRASSICACEAE
***Brassica oleracea* var. *capitata* L.** zelje, zele, zielje, zelce, kapuci (cabbage)
SYM: *Poverty* "Last year I was full of bacon, this year I am full of cabbage!" (Š8450); *Human characteristic* hard (stupid) as cabbage stem (Š2926)
USF: *Consumption*

***Brassica rapa* var. *rapa* L.** repa, repca, ripa, riepa (turnip)
SYM: *Human characteristic* white as turnip (Š2546); *Poverty* "...You will eat white turnip, / And sit hungry by the stove…" (Š8351), *Other*
USF: *Consumption*

***Erysimum cheiri* (L.) Crantz** zlati šebenik*, fajglnček (wallflower)
USF: *Decoration* bouquet (Š2183)

***Raphanus sativus* L.** redkev, rethva, povrtnica (radish)
USF: *Consumption*

BURSERACEAE

***Boswellia sacra* Flueck.** bozvelija*, kadilo (frankincense)
SYM: *Religious* gifts offered to the infant Jesus by the Three Wise Men (Š0195)

***Commiphora myrrha* (Nees) Engl.** mira, miera (myrrh)
SYM: *Religious* gifts offered to the infant Jesus by the Three Wise Men (Š4957)

BUXACEAE
***Buxus sempervirens* L.** pušpan, rožca pušpanova, pušpenje, bušpan, bušpen (box)
ENV: "In my court there are three bushes of box, / under each of them is a bird..." (Š5448)
SYM: *sadness, death*
USF: *Handicraft* smoking pipe (Š2905)

CANNABACEAE
***Cannabis sativa* L.** konoplja, konopla, konopwa, konopljica, konoplina (hemp)
SYM: *Human characteristic* comparing hemp plants to young girls, virgins (Š5081); thin and/or tall as hemp (Š5451)
USF: *Textile*

CARYOPHYLLACEAE
***Dianthus caryophyllus* L.** nagelj, nagel, nageljn, nageln, nagelenj, nagelček, nagelč, nagelnj, nagelnc, naglič, nagelčac, nageljče, nagalenj, naglčk, nagoln, nagolč, nankoln, najgeln, najglnček, nahel, nahelc, najgel, fajgln, fajdel, fajdeln, fajgel, fajl, klinček (carnation)
SYM: *Human characteristic* white carnations as breasts (Š53); lips red as carnations (Š1840); *Female beauty; Male beauty*; *Love* bouquet (Š1117); *Sad love* girl will give a bouquet to her boy who is going away (Š1592); *Sadness*: "Little bird / don't sing to me / all my flowers have frozen / and so has my carnation" (Š4444); *Religious* virgin Mary collecting carnations for st. Joseph (Š4750); angels (Š4907); a virgin who was collecting carnations gave birth to St. Joseph (Š5072); *Other*
USF: *bouquet* (Š61); *Decoration* carnations hanging from window sills (Š1010); *Decoration at funeral* "If I will die / I will have a beautiful wreath / made of gentle rosemary / and red carnations wreath made of carnations... " (Š6250)

COMPOSITAE
***Artemisia absinthium* L.** pelin, pelinek, pelinc (absinthe)
SYM: *Sadness, death* comparing aching heart to a garden of wormwood (Š0234); planting wormwood on a grave (Š0254); young girl must marry an old man because he carries wormwood behind his hat (Š0866), last year I weeded rosemary / this year I weed only wormwood (Š2282); "…Wormwood, wormwood, / You bitter flower, / I will pick you, / And put you around my heart. / Where my love is walking, / Rosemary fruits there, / Green rosemary, / My darling." (Š1428)

***Helianthus tuberosus* L.** topinambur*, laška repa (Jerusalem artichoke)
SYM: *Poverty* "You give me Jerusalem artichoke, / while in the tavern I eat a delicious chicken" (Š6118)
USF: *Consumption*

***Helichrysum* sp.** smilj*, smiljka (immortelle)
USF: *Decoration*

***Lactuca sativa* L.** solata, solatica, šelatica, sovata (lettuce)
USF: *Consumption*

***Santolina chamaecyparissus* L.** nemški rožmarin, rožmarinček nemški, rožmarin laški (cotton lavender)
SYM: *Death* funeral wreath or bouquet made of German rosemary and carnations (Š1194); She was picking up flowers, / to make a bouquet / of German rosemary and red carnation: / "I will wash it with tears, / and tie it with a black thread." (Š61); *Farewell* girls giving their loved ones bouquets of German rosemary and carnations when they go abroad to work or at war (Š1592); *Love* bouquet made of German rosemary and carnations (Š1378)
USF: *Decoration* funeral wreaths, bouquets

***Taraxacum officinale* (L.) Weber ex F.H. Wigg** regrat*, regrut (dandelion)

SYM: *Human characteristic* "Oh dandelions, why are you so yellow?" / "How shouldn't we be yellow, / when all our officers are mad!" (Š7001)

CORNACEAE

***Cornus* spp.** dren (dogwood)

ENV

USF: *Handicraft* stick "I have a stick / hard as dogwood wood..." (Š8462)

CUCURBITACEAE

***Cucurbita* spp.** buča (pumpkin)

USF: *Other*

CUPRESSACEAE

***Juniperus communis* L.** brin, borovica (juniper)

ENV

SYM: *Bedding* "...Juniper roots are my pillows..." (Š1582)

USF: *Consumption* (Š0988); *Handicraft* hoe (5569)

ERICACEAE

***Erica carnea* L.** spomladanska resa (heath); vries

ENV

FAGACEAE

***Fagus sylvatica* L.** bukev, bukva, bukovje, bukvje, bukvica, bukovc (beech); žir, žirek (beechnut)

ENV

SYM: *Poverty* smoking dried beech leaves (Š8558); *Other* "…Oak is getting married / he will marry a beech…" (Š997)

USF: *Wood* beech wood boards (Š265); *Animal feed* beechnuts for pigs (Š7450)

***Quercus* spp.** hrast, hrastiček, hrastič, hrastička (oak); želod (acorn)

ENV

SYM: *Comparison* boys compared to oaks, girls to hemp: "Three oaks are growing behind the house / three handsome boys are living in the house" (Š4732); *Impossible things* song describing how the snail got its shell; snail was tied to an oak (Š8617); *Other*

USF: *Handicraft* cross made of oak wood (6583); *Animal feed* for pigs (Š7451)

GENTIANACEAE

***Gentiana verna* L.** spomladanski svišč*, spanjšice (spring gentian)

ENV: *Landscape* "Pretty spring gentians were blooming; small birds were singing..." (Š0234)

SYM: *Sadness, death* "Spring gentians faded, they were dry and faded. Mila fell asleep among them; never again did she wake up." (Š0234)

GERANIACEAE

***Pelargonium radens* H.E.Moore** roženkravt, roženkraut, roženkrat, rozenkravt, muškatel (rasp-leaf pelargonium)

SYM: *Sad love* girl carrying a fragrant bouquet of pelargonium (Š1118); girl offering a love bouquet of rosemary, carnation and rasp-leaf pelargonium to her loved one (Š1608); *Religious* entering heaven (Š4915), symbol of the Holy Ghost (Š4921), symbol of Virgin Mary (Š4923)

JUGLANDACEAE

***Juglans regia* L.** oreh, joreh (common walnut)

ENV

SYM: *Poverty* smoking walnut leaves (Š8558); *Other* "A gray falcon is flying over Vlach lands, / Nowhere did he find a tree nor a stone, / Only one seed, a walnut seed..." (Š0851)

USF: *Consumption* "If the girl from Bovec is not pretty, / Than her pocket is, / It is full of walnuts, / And hazelnuts in between." (Š2866); *Handicraft* house made of walnut hulls (Š1946), door frame (Š3721)

LAMIACEAE

***Ocimum basilicum* L.** bazilika*, bejžolek, bosiljka (basil)

USF: *Decoration; Other*

***Origanum majorana* L.** majaron, majeron, marijon, marjon (marjoram)

ENV

SYM: *Religious* symbol for Mary's throne (Š4919); *Sadness* "Wilt, all my flowers, / Blue ones, red ones and purple ones, / wilt my marjoram, / I won't need it anymore..." (Š1670); This year I planted marjoram, next year I won't plant even nettle... (Š5431); *Love* (Š3909)

***Salvia rosmarinus* Spenn.** rožmarin, rožmarince, rozmarin (rosemary)

ENV

SYM: *Human characteristic* girl compared to rosemary (Š1140); *Love* rosemary growing in the garden, a girl is collecting or talking to plants and thinking of a boy (Š791); "A widow has stinging nettle on her chest, a young girl has rosemary on her chest" (Š1399); *Preventing love* a mother stole her daughter a rosemary braid so sha cannot give it to her boy (Š1366); *Marriage* "…If I bring in my hands (in church) / a beautiful green rosemary / You will see, how I will say / That I marry you!" (Š732); *Freedom* "Grow, grow, rosemary, / beautiful and green, / so is being single / pleasant and happy. " (Š2320); *Memory*; *Sad love*: "Rosmary is drying, / Its colour will change, / My boy is looking forward, / He will meet a new love." (Š2251); dry rosemary as symbol of sadness (Š2315), girl giving a bouquet to a boy who is going away (Š775), girl is carrying a bouquet, a boy wants to smell it but she doesn't let him because she got engaged to another one (Š1054); "My green rosemary, / You are flowering but you don't bear any fruits, / Same as me, Ivan's love, / I love but I can't give birth..." (Š861), girl/boy offering a wedding gift to her boy/girl who is marrying another girl: an orange and a braid of rosemary (Š2201); *Cheating* "I will dig up a garden, / and plant different flowers in it, / also the green rosemary, / to cheat boys with it." (Š2233); *Sadness* "Last year I weeded rosemary / This year I weed only wormwood" (Š2280); "Rosemary, why do you grow, / if I cannot use you? / My son is sleeping next to my heart, / and noone knows about him yet. " (Š1641); *Death* planting rosemary on a grave (Š504), putting rosemary in a grave (Š256), "Grow, grow, rosemary, / Beautiful virgin memory: / Rosemary has a pleasant smell / whether fresh or dry. " (Š841); *Religious* symbol of Jesus (Š4905); *Innocence*; *Other religious* Virgin Mary making a bouquet of rosemary, carnations and white lilies (Š4862); *Other* describing a wedding: "...Who were the musicians? / Four white doves. / What was the wine? / Only rosemary..." (Š495)

USF: To spray home with rosemary (Š205); *Source of income* selling three pots of rosemary to buy a craddle for a baby (Š1517)*; Other*

***Salvia officinalis* L.** žajbelj*, žalbeljn (sage)

SYM: *Sadness* A lady about to give birth asks the messenger to bring bouquets of sage, "jasenje" (possibly *Fraxinus*?) and tiny flowers for her daughter but dies before he is back (Š98)

LAURACEAE

***Laurus nobilis* L.** lovor*, lorber, lorbek, lomber, lorbov, lorbeg (laurel)

SYM: *Forgiveness* sinner kneels for seven years under a (dry) laurel tree until it becomes green (Š0487); *Beauty* beautiful as laurel flower (Š2907); *Old age* giving figs and carobs to girls and laurel to old women (Š7604)

USF: *Medicine* girl refuses to consume laurel as medicine, she prefers her loved one who to heal her: "I don't want to eat laurel, I prefer my boyfriend: / Laurel is bitter, but my boyfriend cools down my heart!" (Š3066); *Gift* laurel, ginger and carnations given as gifts to a girl by a boy (Š1403)

FABACEAE

***Ceratonia siliqua* L.** rožičevec* (carob), rožič (carob pod)

SYM: *Young age* giving figs and carobs to girls and laurel to old women (Š7604)

***Lens culinaris* Medikus** leča (lentil)

SYM: *Despise* "Vlachs - farting peas, shitting lentils!" (Š7718)

USF: *Consumption*; *Animal feed* bear (Š7943)

***Phaseolus vulgaris* L.** fižol, fižou, fižov, fežov, fažov, fažol, fažoj (common bean)
SYM: *Poverty* beans as prison food (Š7397)
USF: *Consumption*

***Pisum sativum* L.** grah, grahnolka (pea)
SYM: *despise* "Vlachs - farting peas, shitting lentils!" Š7718)
USF: *Consumption* (Š8341), *animal food* (Š8039, Š1865)

***Trifolium* spp.** detelja, detela, detelca, detelka, detel, trava detela, trava diteljina, trava detela, trava ditelina, trava diteljina, detelina trava, deteljina trava (clover)
ENV
SYM: *Sins* writing sins with a clover pen (Š1350); *Death* "…Clover grew from his grave / in the evening it was cut / in the morning it was fresh…" (Š587), "clover is growing around (dead) Marko / beautiful clover grass…" (Š902); *Metaphor for life* "There grows green clover / on a green field. / In the morning the mower comes / and mows the clover... / Oh, sinner / the same will happen to you... / In the evening you will lay down in your bed / all healthy and strong / but then death will come to you / and cut you down..." (Š6120)
USF: *Animal feed* horses (Š215)

***Vicia faba* L.** bob (broad bean)
USF: *Consumption*

LILIACEAE
***Lilium candidum* L.** lilija, lilja, lilica, lila, lelja, leljica, lelija (Madonna lily)
ENV
SYM: *Innocence* (Š1021); *Love* (Š1122); *Beauty* girl compared to lily (Š135); *Sadness* (Š1275); *Sad love* rejected boy will plant lilies, roses and carnation into the heart of his loved one so she will remember him (Š1988); *Religious* Angel Gabriel carrying lilies, Virgin Mary smells the lilies and gets pregnant (Š4757), Lilies growing from the grave of two lovers, sometimes lily from male and rose from female or the way round (Š115), Lilies and violets growing from a drop of Jesus blood, they will smell during the holy messes… (Š452); lilies don't grow where Jesus died (Š6583); lilies representing Virgin Mary (Š4896); *Death*; *Other* Heart was burning, wine was blooming / blooming, blooming / like this white lily (Š5552)
USF: *Ornamental*

LINACEAE
***Linum usitatissimum* L.** lan, len (flax)
USF: *Consumption* "Lanena glavica, / to je budanjska hranica!" (Š7886); *Decoration*; *Textile* retting flax (Š3968)

MALVACEAE
***Tilia* spp.** lipa, lipica, lipca, lipka, lipika, sladovica (linden)
ENV
SYM: *Impossible things* when the dry linden will bloom, the sins will be forgiven (Š484), linden started blooming when virgin Mary and Joseph sat underneath (Š4954), linden without roots and leaves producing a fresh shade (Š7768); *Comparison* three green lindens, three yellow girlfriends (lovers) (Š4298); *Other* (Š997)
USF: *Handicraft* cradle made of linden wood (Š1401)

MORACEAE
***Ficus carica* L.** figa, fija (common fig)
ENV: *landscape* (where the figs grow; Š 3162)
SYM: *impossible things* "I climbed the fig tree, on an apple bashing spree, only pears fell free" (Š7790); *Lying* if he lies, I will show him a fig (Š1403), *Young age* (giving figs and carobs to girls and laurel to old women; Š7604), *Better life* I will go to Italy, where figs bloom (Š3161)
USF: *Consumption*

***Morus* sp.** murva, murava (mulberry)
ENV: giving birth under mulberry (Š0173)

OLEACEAE
***Fraxinus excelsior* L.** jesen (ash)
SYM: *Other* song describing different trees getting married to each other: "Ash is getting married: / He is marrying: / the ridged willow..." (Š0997)

***Olea europaea* L.** oljka, vejica oljenca (olive)
SYM: *Religious* virgins bringing olive branches to church as offering to mother Mary (Š0459); *Salvation* A kidnapped girl asks her savior to bring three crosses made of olive wood on Palm Sunday as one of the gifts that will save her from her kidnapper (Š87)

PAPAVERACEAE
***Papaver rhoeas* L.** mak, purpeljica (rdeča), purpölica (common poppy)
SYM: *Color* red cheeks as poppy (Š2883)

***P. somniferum* L.** purpeljica (bela)(opium poppy)
ENV
SYM: *Color* girl as white as poppy's flower (Š1035); *Sadness* "Last year I weeded / Onion and poppy flowerbeds, this year I won't even weed stinging nettle..." (Š2281)

***Papaver* sp.** (poppy)
USF: *Decoration* The girl picked up some poppies and put them behind boy's hat (Š1121)
SYN: *Beauty* "She is white and red as poppy; she must be from the city..." (Š2548), she is as thin as poppy's flower (Š4975); *Impossible things* to make a shirt from poppy flower (Š5120); *Size* heart shrank to the size of poppy seed (Š0268)

PINACEAE
***Abies alba* Mill.** jelka*, jelčica, hojica, hojčje (silver fir)
ENV
USF: *Other*

***Picea abies* (L.) H. Karst.** smreka, smreča, smrečica, smrejčica, smrečje, smrejčca, jalva, jalova (Norway spruce)
ENV
SYM: *Beauty* "How beautiful is a spruce in bloom / like milk and blood" (Š2150); *Poverty* "…My property/house is a spruce"; *Fear* "Last night I went to the village / I got scared / the green spruces / were bowing down" (Š3337); *Sadness* withered spruce (Š5037), *Other* "Hills of Koroška / are my pillows / spruce branches / are my blankets" (Š1564)
USF: *Wood* to make a boat to reach a girl (Š1576), husband beating his wife on a spruce wooden board (Š7937)

***Pinus* sp.** bor, borovje, borawje (zelen, črni) (pine)
ENV: behind the mountain there is a black pine (Š1095), behind the mountain there is a green pine (Š1101)
SYM: *Love* a pine tree and a castle making love (Š1361); *Other*

PIPERACEAE
***Piper nigrum* L.** poper (black pepper)
USF: *Consumption* shop selling pepper (Š8605)

POACEAE
***Avena sativa* L.** oves, ovsek, voves, zob (oat)
SYM: *Poverty* oats husks as payment (Š7361); girl treats a horse nicer than her boyfriend: she feeds the horse with wheat and her boyfriend oats (Š2155)
USF: *Consumption* oat bread (Š2247), *Animal feed* horses (Š7843)

***Hordeum vulgare* L.** ječmen, jačmien (barley)

ENV: rabbit hiding in barley (Š0976), bird singing about barley ripening (Š8048)
USF: *Consumption* bread (Š0292), pastry (Š1795), threshing barley, oats and wheat (Š8359)
SYM: *Poverty* symbol of poor people's food (Š0292), barley porridge as prisoner's food (Š7386); *Other* (boys are made of barley awns (Š8571)

***Panicum miliaceum* L.** proso (proso millet)
SYM: mouse weeding millet (Š0967)
USF: *Consumption* harvesting millet (Š0447), husking millet (Š3968), *Animal feed* chickens (Š5324), birds (Š0987)

***Secale cereale* L.** rž, hrž, arž (rye)
USF: *Consumption; Bedding* making love in rye straw (Š1930); our bed (in jail) is rye straw (Š7385)

***Triticum aestivum* L. em. Fiori & Paol.** pšenica, pšenička, pšeničica, pšeničkica, pšenula, pšinica, pšenčaka, šenica, šeničica, všenica, všeničica, všeničica, ušenica, ušenička, šenulka (wheat)
ENV
SYM: *Army* "Is this wheat what I see? No, this is the army" (Š102); *Wealth* wheat bread (292), "Before I didn't want to eat wheat bread, now I have to eat triticale" (Š6744); *Love* wheat cake for a loved one (Š3177), wreath made of wheat as gift for the loved one (Š5383), "...When wheat was flowering / You were mine / Now wheat is harvested / You have another one..." (4734); *Religious* wheat growing from Jesus blood (Š452); wheat representing Jesus (Š5918); "There is no such land as India / They never plow the fields, / But they still harvest wheat / Twice a year…" (Š572); *Fertility* where a bird will drop a wheat grain, the land will become fertile (Š5043), where a pear will fall, wheat will grow (Š5077); *Other*
USF: *Animal feed* horse (Š4), mouse (Š964); *Harvesting or sowing wheat* (Š93); *Consumption* (Š133); *Source of income* selling wheat (Š3163); *Religious* wheat to make sacramental bread (Š4927); *Theft* women stealing wheat to buy wine (Š552); *Other*

***Zea mays* L.** koruza, karuza (corn)
USF: *Consumption* (Š7452)

POLYGONACEAE
***Fagopyrum esculentum* Moench** ajda, ajdna, hajda, ejdica, hojda (buckwheat)
SYM: *Poverty* buckwheat bread as symbol of poverty (Š32); *Punishment* father giving his lazy son a bag of buckwheat husks (Š7362); *Beauty* face white as buckwheat flower (Š1514)
USF: *Consumption* "In Koroška, in Carniola / Buckwheat is ripening / A girl is mowing it / Her hands are hurting." (Š2732); bread (Š3532), cake (Š7346), gift for the mythological figure Green George (Zeleni Jurij (Š4987)

***Rumex obtusifolius* L.** topolistna kislica*, ščavlje (bitter dock)
SYM: *Ugliness* face green as sorrel (Š2654)

RANUNCULACEAE
***Clematis vitalba* L.** srobot*, skrebut (old man's beard)
USF: *Handicraft* chests (Š5120)

ROSACEAE
***Fragaria* spp.** jagode, jagodica, jahodca (strawberry)
USF: *Consumption* Eating strawberries and hazelnuts in the forest (Š4951)
SYM: *Spring* I wish there was spring, I wish strawberries and violets grew and girls would pick them (Š0956); *Color* red cheeks as strawberry (Š5451); *Sweetness* lips sweet as strawberries (Š1329)

***Malus domestica* Bork** jablana, jablanca, jablanka, jabuka zelenika, drevje jabkovo (apple tree); jabolko, jabuko, jabuku, jabuka, jabolka, jabowko, jabka, jabuka, jabuko, jabuku, jabčica, jabučica, jabelko, jobloko, jabuku (apple fruit)
ENV
SYM *Regret* a Turk was planting apple and pear trees when he was young, but other people are enjoying them now (Š94); *Love* Boy offering half of a golden apple to his mother, father, sister and brother, they all take a larger half, only his girl takes a smaller one (Š1161); *Human characteristic* boy comparing a person to an apple: "red (positive) on the outside, green (negative) (Š1288),

Fertility where the apple falls, land will become fertile (Š5116); girl who cannot bear a child is compared to a green apple (Š861); *Religious* gift for Virgin Mary (Š4867); apple as symbol of Jesus (Š4906); Virgin Mary lays under an apple tree, the tree starts to bloom (Š4944); wherever Jesus touches with a golden apple, the land there becomes fertile (Š5042); *Impossible things* …until a maple tree will fruit apples… (Š880)

USF *Consumption* a dying soldier is asking his companions to plant an apple at his grave; whoever will be hungry and will pass by the grave will take an apple and say to God to have mercy on his soul (Š261); *Animal feed* for pigs (Š7455); *Other* girl was peeling an apple and throwing the peels at a boy so he would look at her (Š2158); to play with apples (Š5120);

***Prunus avium* L.** češnja, češna, črešnja, črišnja, črejšna (sweet cherry)
ENV: cherries ripening on St. Vitus day (Š7223)
SYM: *Doing something without effort* "We will fight the Turks like we were eating cherries" (Š19); *Love* bringing cherries to the loved one (Š3158); climbing on a cherry and observing a loved one (or cheating one) from distance (Š3782); *Describing seasons* Here the cherries are ripening / there they are still in bloom (Š4669); *Other*

***Prunus cerasus* L.** višnja, višnica (sour cherry)
SYM *Other* (Š5078)
USF: *Consumption* sour cherry as symbol of gift for young lover (Š1069); making shoes from wood (Š5120)

***Prunus domestica* L.** sliva, slivica, češpelina, cimbrov, čvošpeln (common plum)
ENV
SYM: *economic status (richness)* "Herod, the king / Has many plums, / Oh he is so wicked / He doesn't give any..." Š4820; *love* (Š3263-65)
USF: *Other* "plant a plum tree / on my grave / he who walk hungry pass by, / can pick a plum..." (Š0261)

***Prunus persica* (L.) Batsc** breskev*, breskva (peach)
USF: *Consumption* (Š8341)

***Prunus spinosa* L.** črni trn*, terninica, trninica, ternjiče, trnjič, trnenica (blackthorn)
ENV
SYM: *Sad love* "My heart is sad... /.../ Planted with artemisia / Fenced with blackthorn: / My loved one planted it / My loved one fenced it..." (Š2349); *Human characteristic* eyes black as blackthorn (Š5451)

***Pyrus communis* L.** hruška, gruška, ruška (pear)
ENV
SYM: *regret* a Turk was planting pear trees when he was young, but other people are enjoying them now (Š94); *Beauty* comparing a beautiful woman to a ripe pear (Š845); *Love* choosing between a pear and a loved one (Š1171); *Impossible things* pears that fall from the pear tree make the ground fertile (Š5076), when a dry pear tree will flower, the daughter will return home (Š5199), I climbed a fig tree, on an apple bashing spree, only pears fell free (Š7790); *Other* Everyone recognizes a man from Tunčanj / he is carrying a pear in his hand (Š8543)
USF: *Consumption* children stealing pears (Š7544); *Source of income* selling pears (Š7733)

***Rosa* spp.** vrtnica, vertnica, šipkovina, šipek (rožica), roža, rožen cvet, gartroža, gartrož, galtroža, gatroža, gajtroža, gartreža, gavtroža, katroža, gajtrožč, galtrožica, gatreža, gavtroža, gartrež, hartroža (rose)
ENV: Snow is melting in the mountains, and roses are flowering (Š629), "Oh green roses / don't grow close to the path / ... / Boys have threatened, / that they will pluck you / and put you in their hats /..." (Š5033);
SYM: *Love* girl carries a lily, arose and a carnation on her chest; the boy says he will pick them up (Š1122); *Sad love* rose growing from a boy's grave, a lily from girl's (or opposite); they grow in the air and they intertwine (Š727); a boy will plant three white flowers in a girl's heart: a white rose represents old age (Š1989); *Religious* representing Virgin Mary (Š554); representing God (Š4934); representing St. Joseph (Š4948); Virgin Mary making a bouquet of roses, lilies and carnations for St. Joseph (Š4757); roses growing from St. Isidore's grave (Š589), angels offering Virgin Mary a red rose (Š4864); *Human characteristic* lips red like roses (Š369), white cheeks as a (white) rose (Š1329), "*A heart will burn, / a cheek will bloom, / like a rose…*"(Š5549); *Other* comparing roses and lilies to grape: "There is no such flower / as this beautiful rose: / it flowers, and then dries, / but bears no fruit." (Š5413).

USF: *Bouquet* "Rose and rosemary, / I make you a bouquet / a carnation is also red / boys like it!" (Š2405)

RUBIACEAE
***Coffea arabica* L.** kava*, kofe, kafa (coffee)
SYM: *Love* "One cup of coffee / inside some sugar / so my loved one / will open the door for me…" (Š1539)
USF: *Consumption*; *Other* wife sells coffee and sugar to make her husband angry (Š8255)

RUTACEAE
***Citrus sinensis* (L.) Osbeck** pomaranča, pomoranča, naranča, pomarankica, narančica (sweet orange)
ENV: "A bird is singing / in a beautiful green orange tree..." (Š0951)
SYM: *Richness* "What will we eat, my traveller? "White buns and oranges: are you coming with me?" (Š1325); *Other* orange falls on a girl's head and wakes her up from a pleasant dream (Š4932)
USF: *Consumption*

SALICACEAE
***Populus tremula* L.** trepetlika (aspen)
SYM: *Other* maple marrying aspen (Š0997)

***Populus* spp.** topol (poplar)
ENV: "I have a house behind the poplar..." (Š1013)

***Salix* spp.** vrba, verba, verba gerbovača, rakita (willow)
ENV
SYM: *Other* "Ash is getting married / he will marry a willow tree…" (Š997)
USF: *Handicraft* pick made of willow wood (Š5569)

SAPINDACEAE
***Acer campestre* L.** maklen*, klen (field maple)
SYM *Other* field maple marrying linden (Š0997)

***Acer* sp.** javor, javorek, jabor (maple)
ENV: *Other* maple country (Š0947)
SYM: *Impossible things* "You will not merry until maple will bear apples..." (Š0880), "until a dry maple will become green again..." (Š0859);
USF: *Handicraft* violin (Š70), flute (Š0922), cradle (Š2253), arc (Š3722), barrel (Š5120), stick (Š7840)

SOLANACEAE
***Nicotiana tabacum* L.** tobak (rauhtabak, čiktabak) (tobacco)
SYM: *Economic status* "I went to Celovec / I did not eat or drink anything / only smoked and chewed tobacco" (Š7345)
USF: *Consumption*

***Solanum tuberosum* L.** krompir, kromper, kronpier, (potato)
SYM: *Poverty* black/small potatoes as prison food (Š7397); *Other*
USF: *Consumption*

ULMACEAE
***Ulmus* spp.** brest*, brestovje (elm)
ENV: "Elms are growing in front of the house" (Š8555)

URTICACEAE
***Urtica dioica* L.** kopriva, kropiva, kropliva, koprivje, koprivenca (stinging nettle)
ENV: "Grow, grow, stinging nettle, and cover the field with green... " (Š4954)

SYM: *Sadness* Describing what was changed since she got a baby: "Last year I was sewing/weeding out marjoram / this year I won't be sewing/weeding out even stinging nettle" (Š2284), "Last year I gave you a flower bouquet, this year you won't receive one made of nettle" (Š4461), "I wash myself with tears, I dry myself with nettle" (Š211); *Religious* gift for virgin Mary (Š459); *Infanticide* throwing a baby in stinging nettle (Š171)
USF textile (Š193); wreath, bouquet (Š5109)

VIOLACEAE
***Viola* spp.** vijolica, viola, fijolica, fiolica, fejolica (violet)
ENV
SYM: *Love* "Sleep, my flower, sleep, my violet..." (Š8182); *Religious* Jesus asking his mother to bring one of his drops of blood in the wood, where violets and lilies will grow from his blood (Š452); *Spring* "Snow has fallen... Black bird is sitting on the church's roof, I wish there was spring, I wish there were strawberries, strawberries and violets growing..." (Š956)
USF: *Decoration* bouquet (Š2268), violets grown in pots (Š1104)*; Animal feed* (Š870)

VITACEAE
***Vitis vinifera* L.** trta, terta, (vinska) trta, (vinska) tertica, vinski trst, vinenska trtica, vinski ters, vinena jagoda, vinska jagoda, vinska rozga, tersek, terta, ters, trs, tartica, tarta rozga, trta muškoteljna, grizdek vinarski, grozdje, grojzdje, gruozdje, grojzdič, grozdek, grozdič (grape vine)
ENV
SYM: most references to grapes are either worshiping wine and grapes or are related to Religious. *Size* tears as large as grapes (Š15), drops of sweat as big as grapes (Š6579); boy sells his girl for some grapes Š2152-3; grapevine growing from Jesus blood (Š455); *Other religious* Virgin Mary smells grapes and becomes pregnant (Š4750); Jesus blood (Š4927); *Fertility* where a bird will drop a grape, the land will become fertile (Š5043); where a pear/apple will drop, vineyards will grow (Š5077); *worshiping the vine* (Š5413)
USF: *Consumption* wine (Š914), grapes (Š955); *Gift* for a newborn child (Š867), for a loved one (Š868), for a mother (wreath made of grape branches; Š5383); *Source of income* (Š7227); *Other*

ZINGIBERACEAE
***Zingiber officinale* Roscoe** ingver*, imbrek (ginger)
USF: *Gift* boy offering a girl ginger, laurel and carnation (Š1403)
