# Supplementary material for: “I Climbed a Fig Tree, on an Apple Bashing Spree, Only Pears Fell Free”: Economic, Symbolic and Intrinsic Values of Plants Occurring in Slovenian Folk Songs Collected by K. Štrekelj (1895–1912)
Source: Plants (Basel). 2022 Feb 7;11(3):458. doi: 10.3390/plants11030458 (PMC8838053; doi:10.3390/plants11030458)
Supplement: Supplementary file 1 [file plants-11-00458-s001.zip › plants-1580314/plants-1580314 - Appendix Table S2.pdf]

plants

MDPI

Supplementary Materials

Table S2. Table showing the number of plant citations for each plant taxon within each (sub)category.

Scientific name	ENVIRONMENT	USE - HANDICRAFT	USE - DECORATION	USE - CONSUMPTION	USE - ANIMAL FEED	USE - OTHER	SYM - APPEARANCE	SYM - ECONOMIC STATUS	SYM - LOVE	SYM - RELIGIOUS	SYM - SADNESS, DEATH	SYM - OTHER
Abies alba	3	1								1		1
Acer campestre												1
Acer spp.	22	5				1			4			2
Allium ampeloprasum				1								1
Allium cepa				2						1		3
Alnus spp.	2	3										1
Artemisia spp.			1				1				9	1
Avena sativa		1		16	1			1		1		
Berberis vulgaris							1					
Betula pendula	4	3					2		2			1
Boswellia sacra										8		
Brassica oleracea var. *capitata*	1			19			1	14				
Brassica rapa var. *rapa*				13			2	3				
Buxus sempervirens	4	1									1	
Cannabis sativa	2	1					12					4
Carpinus spp.	1											1
Ceratonia siliqua				1								1
Citrus sinensis	1			4					1			
Clematis vitalba		1										
Coffea arabica				12		1			3			
Commiphora myrrha										9		
Convallaria majalis	1											1
Cornus spp.	2									1		2
Corylus avellana	6	2		10	2	2			2	1		
Daucus carota	1			21				1		1		1
Dianthus caryophyllus	1		40				19		22	22	30	4
Erica carnea	3											
Erysimum cheiri			1									
Fagopyrum esculentum				16		2	1	5		3		1
Fagus sylvatica	23	1		1	7			1				3
Ficus carica			2	1								2

Foeniculum vulgare				1								
Fragaria spp.				3			2					
Fraxinus excelsior											1	1
Gentiana verna											1	
Helianthus tuberosus				1				1				
Helichrysum spp.	2											
Hordeum vulgare		1		8		1	1	2				
Juglans regia	2	1		3				2				6
Juniperus communis	2	2		1	1			1		1		
Lactuca sativa				12				3				
Laurus nobilis	1			3			1			1		1
Lens culinaris				5	1		1	2				1
Lilium candidum	2		13				9		1	72	17	2
Linum usitatissimum	1	1		1								
Malus domestica	16	1	1	9	6	8	9	3	12	14	2	8
Morus spp.	2											
Nicotiana tabacum				30				13				
Ocimum basilicum	1		5							5		
Olea europaea										2		
Origanum majorana	1								4	9	1	10
Panicum miliaceum				4	6			1		1		
Papaver rhoeas							1					
Papaver somniferum							6			1		1
Papaver spp.			1				3		1			1
Pelargonium radens			1						6	3	1	
Pelargonium spp.			1								1	
Phaseolus vulgaris				12				9				
Picea abies	6	8							1		2	8
Pinus spp.	19								4			
Piper nigrum						1						
Pisum sativum				3			1					
Populus spp.	1											
Populus tremula												1
Prunus avium	3			6			1		1			1
Prunus cerasus			1	2					3			1
Prunus domestica	2		1							1	1	
Prunus persica				1								
Prunus spinosa	2						2		1		2	
Pyrus communis	10			8		3	2	2	1	3		7
Quercus spp.	12	1			4		3					5
Raphanus sativus				3								1
Rosa spp.	9		7				8		4	63	6	5
Rosmarinus officinalis	1		39	1		2	13		59	44	36	5

Rumex obtusifolius							1					
Salix spp.	5	2								1		1
Salvia officinalis											1	
Sambucus nigra		2					1					
Santolina chamaecyparissus			27				4		2		25	
Secale cereale				3		2		2				
Solanum tuberosum				13								1
Sphagnum spp.						1						
Taraxacum officinale							1					
Tilia spp.	88	3		1		3			2	4		4
Trifolium spp.	11			1	8					1	8	
Triticum aestivum	9	1	1	131	19			12	2	53		
Ulmus spp.												1
Unclassified fern	3			3		5		4	3	3	6	5
Unclassified moss	3						1					
Unclassified Poaceae	8	1		2	2			5		25		1
Unidentified seed plant				1	1					1		
Urtica dioica	2		2							2	6	5
Vicia faba				5				1				
Vinca minor										1		
Viola spp.	2		7						3	1	7	2
Vitis vinifera	17		2	152			8	12	13	104	1	2
Zea mays				2	1							
Zingiber officinale				1								
Sum subcategory		28	152	497	58	31	99	82	151	453	155	101
Sum category	281			766						1041		
Total species	39	15	18	41	12	12	24	20	23	31	20	36
